# A systematic review of physiological methods in rodent pharmacological MRI studies

**DOI:** 10.1007/s00213-014-3855-0

**Published:** 2015-01-15

**Authors:** Jennifer X. Haensel, Aisling Spain, Chris Martin

**Affiliations:** Department of Psychology, University of Sheffield, Western Bank, Sheffield, S10 2TP UK

**Keywords:** phMRI, Cerebral blood flow, Anesthesia, Neuroimaging, fMRI, Pharmacological magnetic resonance imaging, Animal model, Systematic review

## Abstract

**Rationale:**

Pharmacological magnetic resonance imaging (phMRI) provides an approach to study effects of drug challenges on brain processes. Elucidating mechanisms of drug action helps us to better understand the workings of neurotransmitter systems, map brain function or facilitate drug development. phMRI is increasingly used in preclinical research employing rodent models; however, data interpretation and integration are complicated by the use of different experimental approaches between laboratories. In particular, the effects of different anaesthetic regimes upon neuronal and haemodynamic processes and baseline physiology could be problematic.

**Objectives:**

This paper investigates how differences in phMRI research methodologies are manifested and considers associated implications, placing particular emphasis on choice of anaesthetic regimes.

**Methods:**

A systematic review of rodent phMRI studies was conducted. Factors such as those describing anaesthetic regimes (e.g. agent, dosage) and parameters relating to physiological maintenance (e.g. ventilatory gases) and MRI method were recorded.

**Results:**

We identified 126 eligible studies and found that the volatile agents isoflurane (43.7 %) and halothane (33.3 %) were most commonly used for anaesthesia, but dosage and mixture of ventilatory gases varied substantially between laboratories. Relevant physiological parameters were usually recorded, although 32 % of studies did not provide cardiovascular measures.

**Conclusions:**

Anaesthesia and animal preparation can influence phMRI data profoundly. The variation of anaesthetic type, dosage regime and ventilatory gases makes consolidation of research findings (e.g. within a specific neurotransmitter system) difficult. Standardisation of a small(er) number of preclinical phMRI research methodologies and/or increased consideration of approaches that do not require anaesthesia is necessary to address these challenges.

**Electronic supplementary material:**

The online version of this article (doi:10.1007/s00213-014-3855-0) contains supplementary material, which is available to authorized users.

## Introduction

The application of functional magnetic resonance imaging (fMRI) techniques to study effects of pharmacological challenges on brain processes has been termed *pharmacological MRI* (phMRI, Leslie and James 2000). This technique relies on the blood oxygen level dependent (BOLD) contrast mechanism to create functional activation maps using the magnetic susceptibility properties of endogenous deoxyhaemoglobin (Ogawa et al. 1990). Specifically, areas of neural activity are associated with an increase in local cerebral blood flow (CBF) to meet oxygen demands, giving a change in deoxyhaemoglobin levels which is detected by MRI. Importantly, areas of activation are only indirectly determined by measuring haemodynamic changes which are assumed to be coupled to neural activity, including cerebral blood flow, volume and oxygenation (Logothetis 2008).

Pharmacological MRI usually exploits either the BOLD technique or alternately MRI measurements of cerebral blood volume using an administered contrast agent (CBV-MRI) to elucidate the workings of neurotransmitter systems and study mechanisms of drug action on a neurobiological level (Leslie and James 2000; Honey and Bullmore 2004; Borsook et al. 2006; Martin and Sibson 2008; Minzenberg 2012). It thus provides a promising approach particularly in neuropharmacological research areas such as drug development and testing (Tracey and Wise 2001; Borsook et al. 2006; Bifone and Gozzi 2012), and improved MRI system affordability and accessibility suggest that phMRI will be an increasingly important method for future research. As such, the potential limitations of phMRI need to be acknowledged. Whilst much phMRI is conducted in human subjects, the technique is also increasingly used in preclinical research using animal models. A significant factor in preclinical phMRI studies is the use of general anaesthesia in animal studies to avoid movement artefacts during image acquisition or to prevent suffering when additional invasive procedures are concurrently introduced, for instance, to allow multimodal recording (see Steward et al. (2005)). This can be problematic since the administered anaesthetic is itself a neuromodulatory agent which affects basal cerebral haemodynamics, vascular reactivity and/or cerebral metabolism in complex ways (Peeters et al. 2001; Sicard et al. 2003; Masamoto et al. 2009). Additionally, the extent to which these factors are affected is agent specific, i.e. basal values are altered depending on the choice of anaesthetic. Since BOLD signals are necessarily interpreted as a change relative to baseline, such anaesthetic-induced differences can ultimately affect the responses to the drug under investigation. Indeed any parameter changing the animal’s physiological steady state, e.g. blood pressure (BP), respiratory rate, body temperature, potentially modulates the haemodynamic phMRI response to pharmacological challenges (Steward et al. 2005).

Various anaesthetics have been used for phMRI purposes, with each agent having its advantages and disadvantages. Alpha-chloralose is popularly used in functional MRI to achieve robust responses to sensory stimuli as a result of its minimal effects on CBF, respiration and cerebral glucose metabolism compared to other anaesthetics (Ueki et al. 1992; Lindauer et al. 1993; Nakao et al. 2001; Austin et al. 2005). However, the necessity to use a different anaesthetic for induction purposes potentially influences basal physiology in more complex ways. Urethane is also widely used in preclinical neuroscience research and can be an appropriate choice to study neurotransmitter systems due to its modest actions on several receptor types (Maggi and Meli 1986; Hara and Harris 2002). But lowering of heart rate and BP, as well as hyperventilation are disadvantages of urethane, the use of which is also discouraged due to its carcinogenic nature (Field et al. 1993). Common volatile anaesthetics including isoflurane and halothane are more suitable choices for longitudinal studies and additionally have fast induction and recovery rates (Sakai et al. 2005). However, such volatile agents significantly alter baseline CBF independent of neural activity (Hendrich et al. 2001; Masamoto et al. 2009). Respiration rate, BP and cerebral metabolic rate of oxygen (CMRO_2_) are depressed in isoflurane compared to awake conditions (Todd and Drummond 1984; Sicard et al. 2003). Overall, neuroimaging measurements are potentially confounded via complex mechanisms of action of anaesthetics and their (neuro)physiological effects.

Caution is thus required when interpreting preclinical phMRI data, where observed signal changes may reflect an interaction of the effects of the anaesthetic agent and those of the drug under study. Indeed several studies have presented differential responses to pharmacological challenge under varying anaesthetic regimes (Gordon et al. 1995; Gozzi et al. 2008; Du et al. 2009; Liu et al. 2012; Hodkinson et al. 2012). Intravenous cocaine, for instance, evoked CBF increases in alpha-chloralose-anaesthetised rats, but reduced CBF under isoflurane (Du et al. 2009). Similarly, ketamine decreased activity in cortical and hippocampal areas under isoflurane, while alpha-chloralose showed the opposite effect of activation in the cortex and hippocampus (Hodkinson et al. 2012). Administration of levo-tetrahydropalmatine (l-THP) showed least activated areas with isoflurane, most negative activations with urethane and mixed positive and negative activations with medetomidine (Liu et al. 2012). In light of these challenges, this paper will systematically review research methodologies used in preclinical phMRI, particularly with respect to anaesthetic regimes and other physiological maintenance parameters which are likely to impact upon phMRI signal measurements. The review will be restricted to research involving rodents, which are by far the most widely used species in this preclinical field.

## Methods

### Search strategy

The search for eligible articles was conducted in PubMed and Web of Science, with the last search carried out on 25th November 2013. Keywords included “phMRI”, “pharmacological MRI”, “pharmacological fMRI”, “pharmacological functional magnetic resonance imaging”, “phfMRI”, “pharmacological magnetic resonance imaging” and “pharmacologic MRI”. Using Web of Knowledge, the search strategy was supplemented by checking references and citations of each eligible study to identify additional papers. All listed titles and abstracts were first screened for inclusion criteria, and full texts of potential articles were then retrieved to evaluate against eligibility criteria. No restrictions were set on publication dates, but papers had to be published as full articles in peer-reviewed journals. Abstracts, poster presentations, conference proceedings, book chapters, or grey literature were not searched.

### Inclusion and eligibility criteria

To be considered in this review, studies had to use rodents as subjects (specifically rats or mice); phMRI studies investigating humans or animals other than rats or mice were excluded. The methodology section of each paper was required to provide details of the anaesthetic regime during the period of data acquisition, including type and dose of the anaesthetic agent. Similarly, name and dose of the drug under investigation had to be stated, and pharmacological challenge needed to occur under anaesthesia. Given the nature of this review, animals had to be anaesthetised during data acquisition, with haemodynamic-based MRI sequences used (e.g. BOLD, CBV, CBF readouts). Studies were discarded if only anatomical MRI or non-MRI neuroimaging methods (e.g. PET) were used. A study was considered ineligible if event-related sensory stimulation protocols were used prior to or during pharmacological challenge (e.g. forepaw stimulation; Kida et al. 2006) but was included when stimulation occurred after phMRI data were acquired or in a separate experimental group. Articles were also excluded if the same drug was tested under several anaesthetic agents or varying doses of anaesthesia (e.g. Liu et al. 2012). Finally, studies which explicitly stated that pharmacological challenge was applied to purposely change basal physiological state were excluded (e.g. norepinephrine for hypertension; Wang et al. 2006).

### Data recording

Factors of interest for this review were identified prior to searching for eligible studies. These included information on citation (author, date, journal), subjects (strain, sex, weight, number of animals), surgical preparation (tracheotomy/intubation for mechanical ventilation, blood gases, if applicable), anaesthetic regime during induction and maintenance (agent, dose, additional gases if applicable), physiological measurements (body temperature, BP, heart rate), pharmacological challenge (drug type, dose, targeted molecular and/or neurotransmitter system if stated) and phMRI method.

## Results

We identified 126 eligible studies for review, and a complete list of reviewed papers is included in the Supplementary References. A summary of the details of each included study is shown in Table S1. Most studies investigated rats (92.1 %), with the most common strain being Sprague–Dawley rats (57.9 %), while 7.9 % used mice as subjects. The use of male rodents was also more prevalent (81.7 %) than females (4.8 %; 12.7 % not reported, 0.8 % used both males and females). Ten studies (7.9 %) reported both the age and the weight of animals in the study, 68.3 % reported weight alone and 6.3 % reported age alone; 17.5 % of studies gave no indication of animal age or weight. A list of journals in which papers were published is presented in Table S2.

### Anaesthetic regime for induction

**Fig. 1:**
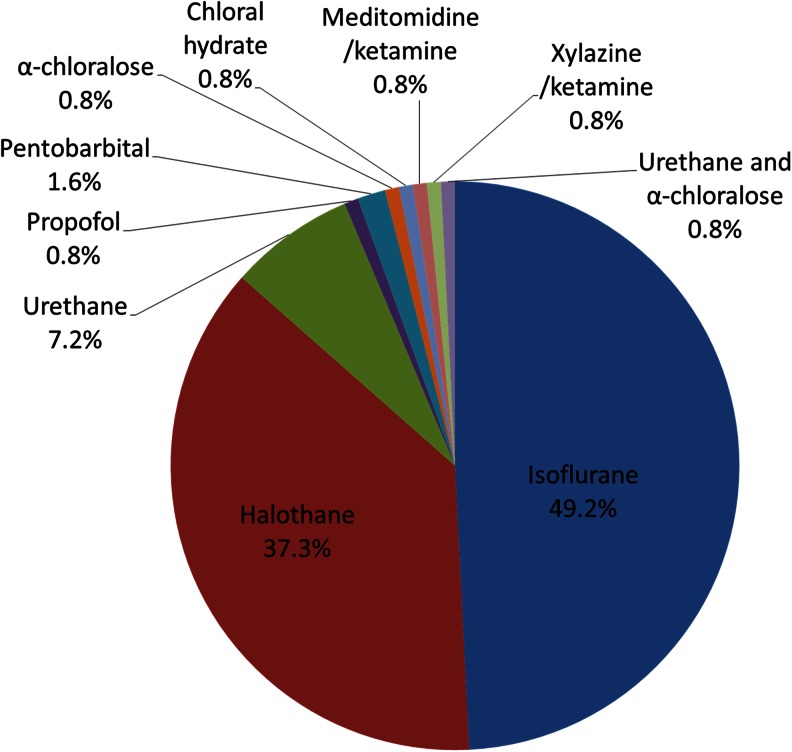
Choice of anaesthetic agent for induction, by percentage of studies

For induction purposes, the volatile agent isoflurane was the most commonly employed anaesthetic agent (49.2 %), followed by halothane (37.3.2 %; see Fig. 1). Urethane was used in 7.1 % of studies, pentobarbital in 1.6 %, and chloral hydrate, propofol and α-chloralose were used in one study each. Ketamine was used in two studies, each time in combination with a different drug—either xylazine or meditomidine. One study reported the combined use of urethane and α-chloralose.

### Anaesthetic regime for data collection and ventilatory gas mixtures

**Fig. 2:**
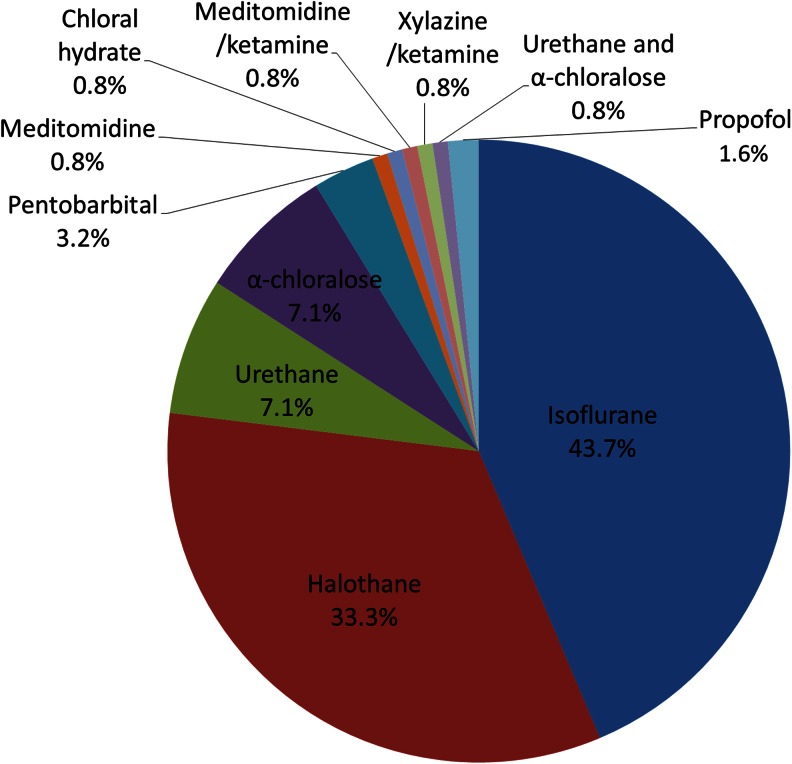
Choice of anaesthetic agent during maintenance, by percentage of studies

Twelve studies (9.5 %) changed the type of anaesthetic agent for maintenance after initial induction, of which eight studies (6.3 %) used alpha-chloralose (which is unsuitable for induction), two used pentobarbital (1.6 %), and individual studies used propofol (0.8 %) or medetomidine (0.8 %). The majority of studies continued with isoflurane (43.7 %) for maintenance, closely followed by halothane (33.3 %; Fig. 2). In total, α-chloralose was used in 7.1 % of studies, pentobarbital in 3.2 %, propofolin in 1.6 % and chloral hydrate in one study (0.8 %). One study used a meditomidine/xylazine mixture for maintenance. Studies using urethane alone (7.1 %), urethane in combination with α-chloralose (0.8 %) or a xylazine/ketamine mixture (0.8 %) require only a single dose of anaesthetic for both induction and maintenance.

**Fig. 3:**
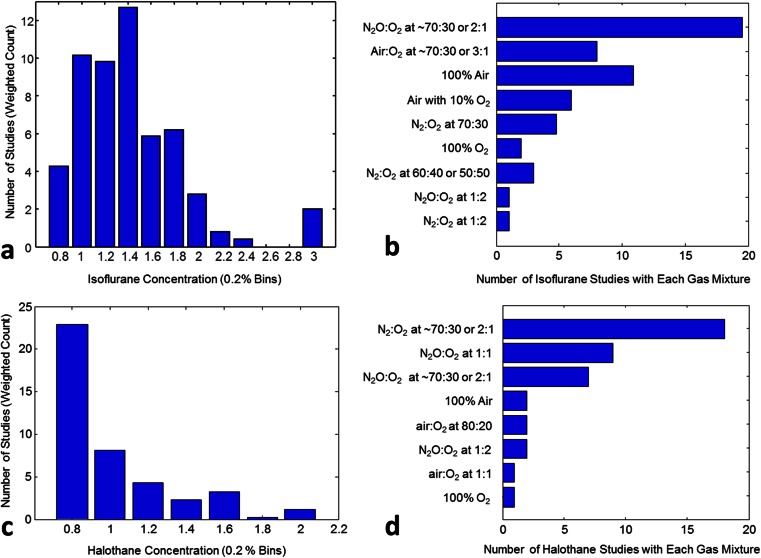
Gaseous anaesthetic doses and ventilator gas mixtures: **a** variation in concentration within the isoflurane group, **b** variation in mixture of ventilatory gases within the isoflurane group, **c** variation in concentration within the halothane group, **d** variation in mixture of ventilatory gases within the halothane group. For **a** and **c**, the bin width of specified concentrations was 0.2 %, with bin centres denoted on the *x*-axis (e.g. 0.8 % corresponds to concentrations 0.7 ≤ *x* < 0.9 %). For **b** and **c**, very similar specified gas mixtures, such as a “70:30 N_2_O/O_2_ mix” and “N_2_O/O_2_ in a 2:1 ratio” were grouped together

We then explored the anaesthetic and ventilatory gas mixtures for the isoflurane and halothane groups in more detail. Dosage data were counted in 0.1 % bins, grouped in 0.2 % bins for plotting in Fig. 3. Because many studies specified a dosage range rather than a fixed value, for the purposes of constructing the histograms in Fig. 3a, c, we calculated a weighted count, whereby the unitary contribution of a single study was divided by the number of 0.1 % bins in the specified range. For example, a study specifying 1.0–1.2 % would contribute 0.5 to both of the 1.0 ≤ *x* < 1.1 and 1.1 ≤ *x* < 1.2 % bins. Within the isoflurane group, maintenance doses ranged from 0.8 to 3 %, with the modal dosage at ~1.4 % (Fig. 3a). Additional gases complementing isoflurane also varied between studies (Fig. 3b), with an N_2_O/O_2_ regime with different concentrations applied in 34.5 % of isoflurane studies.

Similarly, halothane dosage varied between studies with a range from 0.7 to 2 % (Fig. 3c). The modal dosage was 0.8 %, with these studies originating from the same group of authors. Figure 3d illustrates the differences in ventilatory gas regimes for halothane, with the modal choice being N_2_/O_2_ (70:30 %).

Alpha-chloralose doses ranged from 50 to 80 mg/kg for initial bolus injection, and continuous administration ranged from 26.7 to 40 mg/kg/h. For urethane, a dose of 1.2 g/kg was chosen in seven out of nine studies, three of which applied additional gases (30 % O_2_/air). The remaining two reported a slightly higher dose of 1.75 g/kg. In the four cases of pentobarbital anaesthesia, two reported a 37.5-mg/kg/h dose with additional gases (N_2_/O_2_ at 1:1), one for 10 mg/kg/h and one for 40 mg/kg. Two studies used propofol, with doses of 60 or 35 mg/kg/h and mechanical ventilation with oxygen-enriched air (in a mechanically ventilated mix 7:1 air/O_2_ or 1:1 air/O_2_, respectively). Each of the following anaesthetics were used in only one study: chloral hydrate 500 mg/kg, then 150 mg/kg; ketamine 0.1 ml/10 g of 2.5 % xylazine and 15 % ketamine in 82.5 % PBS at 0.1 ml/10 g/h; mixed 1 g/kg urethane with 65 mg/kg alpha-chloralose; mixed 1 mg/kg medetomidine with 75 mg/kg ketamine.

Overall, the combinations of anaesthetic types, doses and ventilatory gases used meant that across our sample of 126 studies, the largest ‘group size’ of studies using identical or very similar methodology was 18, of which 17 studies included the same authors. In total, there were 73 different anaesthetic/ventilatory gas regimes reported over the 126 studies.

### Ventilation and respiration protocol

Mechanical ventilation was performed in 52.4 % of studies, while 45.2 % did not artificially control animals’ respiratory rates. In three studies (2.4 %), only a subset of animals was artificially ventilated. Of those studies where animals were not artificially ventilated, 54.4 % monitored respiration rates.

### Additional monitoring

Blood gas parameters were measured in 62.7 % of studies, while the remaining 37.3 % did not report recordings. Body temperature was reported as being monitored and controlled in 91.3 % of studies. Approximately two thirds of reviewed studies recorded at least one cardiovascular parameter in the form of heart rate and/or BP, whilst the remaining third did not report any such measurements. Moreover, 56.3 % of studies reported recording blood pressure, and 33.3 % of studies reported recording heart rate during data collection.

### fMRI method

BOLD was the most commonly used phMRI measure, being used alone in 51.6 % of studies or in combination with other techniques in 6.3 % of studies. rCBV measures alone accounted for 36.5 % of studies and in combination with other measures accounted for 6.3 % of studies; 4.8 % of studies measured CBF in isolation, and it was used in combination with other measures in 2.4 % of studies. Studies using combined techniques accounted for 7.1 % of the total number of studies.

## Discussion

The present review of preclinical phMRI methodologies revealed that anaesthetic regimes and related methodology vary substantially between laboratories, with roughly half as many differing approaches as there were research studies in our sample. A range of anaesthetics have been used for both induction and maintenance purposes, most notably the volatile agents isoflurane and halothane. Choice of dosage levels as well as the mixture of ventilatory gases also differed between as well as within research groups administering the same anaesthetic. Studies employing very similar or identical anaesthetic regimes tended to originate from the same laboratories. With respect to physiological monitoring, studies generally reported on such parameters, but variation existed with respect to the type of recorded parameter, and cardiovascular measurements in particular were not always reported. Lastly, roughly half of the reviewed studies used BOLD contrast fMRI, followed by CBV weighted imaging, and a minority performed CBF measurements.

The major effects of the main anaesthetic types reported upon neurotransmitter systems as well as cerebrovascular and systemic effects are summarised in Table 1. Since evidence suggests that phMRI data are likely to be affected by the choice of anaesthetic regime due to differential actions of anaesthetic agents (Gordon et al. 1995; Gozzi et al. 2008; Du et al. 2009; Liu et al. 2012; Hodkinson et al. 2012), the apparent laboratory dependence of these important aspects of research methodology is a major concern. Specifically, interpretation of the neurotransmitter effects of a drug under investigation should consider likely interactions with neurotransmitter effects of the anaesthetic (Table 1), especially when comparing between different studies. Anecdotally, it seems as though many laboratories (our own included) establish and then stick to an animal preparation that works well in their hands for their particular application: this view appears to be supported by the present findings. For fMRI studies, alpha-chloralose is often described as one of the most effective anaesthetic agents due to its minimal effects on basal physiology (Ueki et al. 1992; Lindauer et al. 1993; Nakao et al. 2001; Austin et al. 2005); however, both isoflurane and halothane were the most common choices for the phMRI literature reviewed here, possibly due to the ease of induction, level of control, fast recovery rates (Sakai et al. 2005) and the advantage of reduced adverse effects for animals that are recovered for chronic experimental designs. However, these volatile agents, particularly halothane, are known for their vasodilatory properties, resulting in increased baseline CBF. Given that most neuroimaging signals used in phMRI describe changes relative to baseline, this may result in smaller activation-evoked changes compared to anaesthetics with lesser effects on CBF. To illustrate, Du et al. (2009) found CBF increases in response to cocaine under alpha-chloralose, but CBF reductions under isoflurane. This is consistent with the minimal effects of alpha-chloralose on CBF, thereby retaining CBF values close to basal state and facilitating a relative increase of CBF by cocaine, whilst a higher baseline CBF under isoflurane may has confounded drug effects. A greater reduction of cerebral metabolic rate of oxygen (CMRO_2_), a factor in BOLD contrast phMRI, has also been found, even more so for isoflurane compared to halothane regimes (Todd and Drummond 1984). The known effects of these agents in relation to neurovascular coupling and the interpretation of phMRI data may make experimentally determined drug effects difficult to generalise and synthesise across laboratories and studies.

**Table 1:** Summary of effects of commonly used anaesthetic agents in animal imaging studies on neurotransmitter systems as well as systemic and cerebrovascular parameters

Anaesthetic	Affected neurotransmitter systems and receptors (in CNS)							Systemic and cerebrovascular effects
	GABA	Glutamate	Glycine	Acetylcholine	Serotonin	Norepinephrine	Dopamine	
Isoflurane	Increased Cl^−^ permeability of GABA_A_	Inhibit release, NAMDAR inhibitor, AMPA inhibitor	Enhancement of glycine-mediated inhibition	Neuronal nAChR antagonist, decreases release	Inhibits release, enhancement of 5-HT_3_R action	Regions specific increase in release	Enhancement of DA realease	Vasodilation, hypotension respiratory depression, reduced CMR(O_2_), altered CMR(Glu)
Halothane	Increased Cl^−^ permeability of GABAreceptors	NAMDAR inhibitor AMPA	Enhancement of glycine-mediated inhibition	Neuronal nAChR antagonist, reduction in turnover	Enhancement of 5-HT_3_R action	Region specific reduction in turnover	Elevation of striatal DA levels	Vasodilation, hypotension respiratory depression, altered CMR(Glu)
Urethane	Potentiation of GABA_A_ effects	Inhibit release, NAMDAR inhibitor AMPA inhibitor	Potentiation of glycine receptor effects	Potentiation of neuronal nAChRs	Altered function	Region specific alteration in release	Reduction of striatal DA levels	Hyperglycaemia*, haemoconcentration*, hypotension, bradycardia, toxicity of mesenteric vasculature and abdominal organs*,
α-chloralose	Increased Cl^−^ permeability of GABAreceptors	NAMDAR inhibitor	No effect on glycine receptors	No effect on AChRs	No effect (tested in specific regions)	Region specific alteration in release	No effect (tested in specific regions)	Respiratory depression, metabolic acidosis, and hyperreactivity, decreased cerebrovascular reactivity, altered CMR(Glu)
Pentobarbital	Enhance activity of GABA at GABA_A_	Inhibit release, NAMDAR inhibitor AMPA inhibitor	Potentiation of glycine receptor effects	Inhibit release, blockade of nAChR	5-HT_3_R inhibition	Inhibit release	Region specific reduction in release	Respiratory and cardiovascular depression, decreased CBF, unclear effects on cerebral blood vessels

In addition to the inherent vasodilatory properties of some anaesthetics, neurovascular effects are concentration dependent (Eger 1984; Maekawa et al. 1986; Abo et al. 2004; Masamoto et al. 2009) and region dependent (Farber et al. 1997; Hendrich et al. 2001; Sicard et al. 2003; Thomas et al. 2014). Our results show that levels of isoflurane and halothane used ranged from 0.8 to 3 % and 0.7 to 2 %, respectively. To some extent, variation in the dose of gaseous (and indeed other) anaesthetic agents may be expected to modulate the magnitude of the principal effects summarised in Table 1, with such modulations likely to be non-linear in nature. Even though small differences are not expected to induce large physiological variability, the above values vary greatly in range and are likely to result in very different physiological, neurophysiological and neurovascular effects, again complicating data comparisons between laboratories. Greater resting CBF has been linked to increasing levels of isoflurane (Cucchiara et al. 1974; Masamoto et al. 2009), as seen by a dose-dependent CBF increase in response to single-pulse stimulation for isoflurane levels between 1.1 and 2.1 % (Masamoto et al. 2009). Given the present range in isoflurane levels for maintenance anaesthesia, these variations in dosage may differentially affect neuroimaging results. Schummers et al. (2008) also found that isoflurane concentrations (0.6–1.5 %) dose-dependently reduced responses of astrocytes, whilst neuronal sensitivity was significantly less affected. Given accumulating evidence for the central role of astrocytes in neurovascular coupling (Haydon and Carmignoto 2006; Carmignoto and Gómez-Gonzalo 2010; Figley and Stroman 2011), the mechanism underpinning BOLD fMRI, decreased sensitivity of involved astrocytes as a result of dosage variations could hence affect phMRI data. Further, while isoflurane uniformly increases global CBF, regional CBF is found to be heterogeneous, with less vasodilatory effects in cortical areas relative to subcortical structures (Manohar and Parks 1984; Farber et al. 1997; Li et al. 2013). This again poses a difficulty in inferring upon true drug effects from (typically) whole-brain phMRI data since responses evoked by pharmacological challenge may depend on a three-way interaction of anaesthetic protocol, brain region and administered drug.

phMRI studies also differed substantially in terms of the composition of ventilated breathing gases, with a common combination being oxygen (O_2_) and nitrous oxide (N_2_O). Relatively few studies have investigated the impact of differing inspired gas compositions (of the type found in this study) upon neuronal, neurovascular or haemodynamic processes. For instance, although the effects of hypercapnia and hypoxia have been explored in some depth, little research has been conducted on how normal or supra-normal concentrations of oxygen, or the use of nitrogen vs nitrous oxide, affect these systems in the context of neuroimaging. Sicard and Duong (2005) manipulated inspired O_2_ (and CO_2_) concentrations under isoflurane anaesthesia and found both physiological parameters and relative, i.e. baseline dependent, fMRI responses to electric forepaw stimulation to differ significantly between conditions. Specifically, supranormal inhaled O_2_ concentrations (100 %) altered heart rate, blood O_2_ and CO_2_ concentrations and pH and increased baseline CBF and BOLD signals. Breathing gases have also been found to have agent-specific neurophysiological effects. For instance, no significant difference in CBF was found between 1.0 MAC halothane alone compared to 0.5 MAC halothane with 0.5 MAC nitrous oxide; however, CBF greatly increased with 0.5 MAC isoflurane with nitrous oxide compared to 1.0 MAC of isoflurane only (Hansen et al. 1989). This suggests that ventilatory gases potentially give rise to differential responses to pharmacological challenges when relative fMRI measures are used. Notwithstanding this, where laboratories optimise their ventilatory gases in order to achieve a stable set of (systemic) physiological parameter values, it is likely that the effects of these differences are minimised and may to some extent usefully mitigate against the differing physiological effects of different anaesthetic agents and concentrations.

Other factors which may affect relative fMRI measurements are physiological parameters such as body temperature, respiration and heart rate, blood pressure and blood gas parameters (see Steward et al. 2005). Our results have shown that not all studies have fully reported on these physiological factors despite their potential role in altering imaging results indirectly. We acknowledge the possibility that studies may not have reported on a physiological parameter despite having monitored it (e.g. body temperature), although future studies should aim to explicitly describe animals’ physiological states. Briefly, body temperature can affect fMRI data by changes in oxygen and glucose metabolism if not kept in normal range (Carlsson et al. 1976; McCulloch et al. 1982), and so animals need to be homeothermically maintained. Blood gases should be recorded and respiration rate controlled by mechanical ventilation or at least monitored to enable control for hyper/hypoventilating animals. Heart rate and/or BP should also be continuously recorded since systemic drug effects can arise in terms of cardiovascular changes, as demonstrated by studies administering noradrenaline to induce blood pressure changes which ultimately result in imaging findings which differ from control conditions (Wang et al. 2006; Gozzi et al. 2007). Blood pressure in particular is a useful physiological measure since analysis techniques can correct for cardiovascular effects by modelling the known BP changes and how these may affect brain haemodynamics as a covariate. In addition, the recording of heart rate and respiration rate will assist subsequent research groups in more precisely matching the physiology, including anaesthetic depth, of their animals to previous experiments in order to facilitate comparison of data. However, our review revealed that approximately one third of studies did not monitor cardiovascular parameters.

Other differences between animal models in terms of subject sex, age/weight, strain or species are also important to consider. Although these concerns are ubiquitous in in vivo neuroscience research, they may interact with some of the specific phMRI issues raised here. For instance, oestrous cycles in female rodents have been shown to influence fMRI BOLD signalling (Dietrich et al. 2001), whilst age and associated brain development (which has separate neuronal, vascular and neurovascular trajectories; Harris et al. 2011) may be a critical factor to control for in investigations of a wide range of drug actions. It is therefore important that parameters such as strain and sex are at the very least reported, yet in our sample 12.7 % of studies did not report animal sex and 4.0 % of studies did not report animal strain. Weight, rather than age, was reported in 68.3 % of studies, while 17.5 % of studies gave no indication of animal developmental level. Variability in species or strains used, whilst an additional source of complexity, does however offer some advantage in terms of improving robustness and confidence in research findings that are consistent across different animal models. Indeed the same may be said of inconsistencies in other aspects of methodology such as anaesthetic regime. A comprehensive description of methodology, including subject details, is key to translating the potential challenges in methodological variability into sources of additional confidence in research observations.

The studies reviewed utilised a range of MRI techniques in order to obtain functional maps associated with drug treatment, including BOLD, CBV and CBF weighted pulse sequences. Which of these approaches are most appropriate given the issues outlined above? Although half of the studies reported utilised BOLD imaging, it has a number of limitations. Firstly, the BOLD signal reflects composite changes in CBV and oxygenation that are in turn driven by metabolism and CBF changes, with the latter arising most directly from neurovascular signalling (Martin 2014). The BOLD contrast mechanism thus has additional points of susceptibility to anaesthetic or methodological factors relative to some other neuroimaging modalities. Secondly, BOLD signals provide qualitative rather than quantitative measures of underlying haemodynamic changes, with signals expressed as a percentage change relative to an essentially arbitrary baseline value. Thus, comparisons between studies where haemodynamic baseline parameters may be altered are difficult. For instance, it is not possible to determine whether a reduction in BOLD signalling is due to a reduced drug action or to an elevated baseline BOLD signal upon which equivalent (percentage) increases are limited due to non-linearities in haemodynamic responding (Vazquez et al. 2006). Perfusion-based imaging techniques such as arterial spin labelling, which determines cerebral blood flow changes by measuring the progression of ‘magnetically tagged’ blood, as well as contrast-enhanced CBV weighted imaging (which uses an exogenous contrast agent to enhance signals from blood), are able to provide quantitative measurement of cerebral blood flow and volume changes, respectively. These were employed in a number of the studies reviewed here. Such measures greatly facilitate comparisons between studies, subjects, brain regions, drug or anaesthetic conditions. By combining BOLD imaging with concurrent CBF and/or CBV data acquisition, it is also possible to calculate additional parameters such as the cerebral metabolic rate of oxygen (e.g. see Luo et al. 2009). A more comprehensive measurement of the haemodynamic response simultaneously improves the characterisation of drug effects and possibilities for differentiating these effects from the physiological impact of methodological factors which we show here to vary considerably between studies.

The present review raises awareness for future fMRI-based pharmacological studies. We have highlighted some issues related to the effects of anaesthetics on the basal physiological state of the animal, which could ultimately bias the interpretations of the drug effects under investigation. Differences in anaesthetic regimes combined with unknown interactions between applied anaesthetics and investigated drug complicate comparisons on pharmacological effects. This also has implications for relating findings from animal studies to those from human phMRI: in contrast to rodent neuroimaging, participants are not usually anaesthetised, resulting in major differences in physiological state for each species given the neuromodulatory effects of anaesthetics (e.g. Deakin et al. 2008; see Anderson et al. 2008). Anaesthetic effects thus potentially confound translational predictions for human phMRI, exacerbating the already complex issue of species differences (Markou et al. 2009).

The choice of anaesthetic regime depends on several factors, thus ruling out a single protocol for phMRI studies. It is desirable to employ an anaesthetic which minimally affects basal physiology; however, the neurotransmitter system targeted by the drug under investigation as well as by the anaesthetic also needs to be considered, primarily to avoid actions on the same system which are known a priori. For instance, a drug modulating GABAergic neurotransmission (e.g. anti-epileptic drugs, Treiman 2001; Madsen et al. 2009) may compete with the typical GABAergic actions of some anaesthetics, thereby potentially confounding the insights into drug action. Route and time course of administration is also an important consideration: most of the studies reported here utilise an inhalational agent with either intravenous or intraperitoneal administration of the drug under investigation. It is important to consider the potential differences in pharmacodynamics and effective time course (including stability) of anaesthetic action and how this could interact with equivalent parameters for action of the drug under investigation. Gaseous anaesthetics such isoflurane and some injectables are rapidly metabolised compared to urethane where a single injection can give stable anaesthesia for >10 h. Although the former agents require constant infusion, providing an advantage for rapid modulation of anaesthetic depth, accumulation of the agent in the body (especially in the fat) can produce variations in effective anaesthetic dose over time. This can be an important consideration in phMRI, where this may produce signal changes occurring over the same time frequency domain as those produced by the drug under investigation. Separating slow oscillations in signals arising due to anaesthetic administration, drug action or indeed non-physiological sources such as hardware noise can be a challenging component of data analysis. Control experiments are therefore critical to determine the extent of non-drug signal changes, and in some cases, it may be desirable to conduct pilot phMRI experiments under different anaesthetic regimes before choosing the appropriate type and level of agent (e.g. Asanuma et al. 2008).

Another method which currently receives increased attention due to its advantage of avoiding confounding anaesthetic effects is the development of awake animal models for neuroimaging studies (e.g. Skoubis et al. 2006; Chin et al. 2008; Ferris et al. 2011; Brydges et al. 2013; Martin et al. 2013). Data from these approaches are potentially much more readily translatable human research, where anaesthetics are rarely employed for phMRI studies, as well as to the field of behavioural (neuro)pharmacology. In these approaches, it is however important to avoid substituting the deleterious effects of anaesthesia with alternate complexities introduced by stress where animals are restrained (and due to the acoustic noise of the MRI environment). Although habituating or conditioning animals to tolerate forms of immobilisation to enable imaging is possible, careful monitoring of additional physiological parameters is also important in order to ascertain the effectiveneness of this approach and to ensure that a state of ‘learned helplessness’ has not been induced by the habituation procedures (e.g. see Reed et al. 2013). Given the well-established effects of stress and restraint stress in particular (itself a model for inducing depression in rodents), consideration of these factors is critical in the phMRI context, where interactions of (for example) activation of the hypothalamic adrenal axis with drug challenges may confound data interpretation. Interestingly, a recent study by Scott et al. (2013) describes a promising approach whereby rats are trained by operant conditioning to voluntarily accept head immobilisation for neuroimaging (albeit in a microscopy rather than MR imaging environment). Such approaches, which may be facilitated by developing preclinical MR environments optimised for work in conscious animals, may represent an important route for future development.

This review reveals the extent of variation in research methodologies used for rodent phMRI studies, showing differences not only in choice of anaesthetic agents but, importantly, also in associated dosage levels as well as variations in type and concentration of additional ventilatory gases and physiological monitoring and regulation. To conclude, we make a series of recommendations, based on the findings of this review, for the design and reporting of future preclinical phMRI studies:

1. The anaesthetic regime should be actively chosen primarily on the basis of two criteria: (a) how to minimise possible drug–anaesthetic interactions; (b) facilitation of critical comparisons or synthesis with previous research findings. Authors should provide a brief rationale for choosing a specific anaesthetic regime and consider the impact of this choice on the specific research findings.
2. Physiological parameters, including as a minimum heart and respiration rate, blood gases (O_2_ and CO_2_) and, where possible, blood pressure measurements, should always be provided. This will allow (a) a fuller appreciation of physiological drug effects and (b) standardisation of physiological status between differing anaesthetic regimes/laboratories.
3. Studies should attempt to provide an indication of anaesthetic depth via any available physiological (EEG, ECG) or other (e.g. reflexes) indicators in order to facilitate matching of anaesthetic depth between laboratories and methodological approaches.
4. Consider an additional ‘control group’ of animals or, where possible, a repeated-measures approach, in which either the anaesthetic dose or the anaesthetic type is altered in order to elucidate the impact of these choices upon the data acquired and improve confidence in the main findings.
5. phMRI protocols to acquire more than one haemodynamic measure (e.g. combined BOLD and CBF imaging) should be used routinely since these can be deployed with little cost to spatial or temporal resolution (or experimental duration) yet provide a more comprehensive assessment of haemodynamic responses to drug challenge.
6. Now that methods for conducting phMRI in un-anaesthetised animals have been developed, these should also be given consideration when planning non-invasive preclinical neuroimaging studies.

## Electronic supplementary material

Below is the link to the electronic supplementary material.

ESM 1 (DOCX 41 kb)
