# Supplementary material for: A systematic review of physiological methods in rodent pharmacological MRI studies
Source: Psychopharmacology (Berl). 2015 Jan 15;232(3):489–99. doi: 10.1007/s00213-014-3855-0 (PMC4302233; doi:10.1007/s00213-014-3855-0)
Supplement: Supplementary file 1 — (DOCX 41 kb) [file 213_2014_3855_MOESM1_ESM.docx]

**Supplementary references: references included in systematic review**

Baslow MH, Dyakin V V., Nowak KL, et al. (2005) 2-PMPA, a NAAG peptidase inhibitor, attenuates magnetic resonance BOLD signals in brain of anesthetized mice: evidence of a link between neuron NAAG release and hyperemia. J Mol Neurosci 26:1–15. doi: 10.1385/JMN:26:1:001

Bosshard SC, Grandjean J, Schroeter A, et al. (2012) Hyperalgesia by low doses of the local anesthetic lidocaine involves cannabinoid signaling: an fMRI study in mice. Pain 153:1450–8. doi: 10.1016/j.pain.2012.04.001

Bouet V, Klomp A, Freret T, et al. (2012) Age-dependent effects of chronic fluoxetine treatment on the serotonergic system one week following treatment. Psychopharmacology (Berl) 221:329–39. doi: 10.1007/s00213-011-2580-1

Broberg B V., Madsen KH, Plath N, et al. (2013) A schizophrenia rat model induced by early postnatal phencyclidine treatment and characterized by Magnetic Resonance Imaging. Behav Brain Res 250:1–8. doi: 10.1016/j.bbr.2013.04.026

Brown SL, Ewing JR, Kolozsvary A, et al. (1999) Magnetic resonance imaging of perfusion in rat cerebral 9L tumor after nicotinamide administration. Int J Radiat Oncol Biol Phys 43:627–33.

Byun NE, Grannan M, Bubser M, et al. (2014) Antipsychotic drug-like effects of the selective M4 muscarinic acetylcholine receptor positive allosteric modulator VU0152100. Neuropsychopharmacology 39:1578–93. doi: 10.1038/npp.2014.2

Canese R, Adriani W, Marco EM, et al. (2009) Peculiar response to methylphenidate in adolescent compared to adult rats: a phMRI study. Psychopharmacology (Berl) 203:143–53. doi: 10.1007/s00213-008-1379-1

Canese R, Marco EM, De Pasquale F, et al. (2011) Differential response to specific 5-Ht(7) versus whole-serotonergic drugs in rat forebrains: a phMRI study. Neuroimage 58:885–94. doi: 10.1016/j.neuroimage.2011.06.089

Caramia F, Yoshida T, Hamberg LM, et al. (1998) Measurement of changes in cerebral blood volume in spontaneously hypertensive rats following L-arginine infusion using dynamic susceptibility contrast MRI. Magn Reson Med 39:160–3.

Chen YC, Mandeville JB, Nguyen T V., et al. (2001) Improved mapping of pharmacologically induced neuronal activation using the IRON technique with superparamagnetic blood pool agents. J Magn Reson imaging 14:517–24.

Chen Y-CI, Choi J-K, Andersen SL, et al. (2005a) Mapping dopamine D2/D3 receptor function using pharmacological magnetic resonance imaging. Psychopharmacology (Berl) 180:705–15. doi: 10.1007/s00213-004-2034-0

Chen Y-CI, Galpern WR, Brownell A-LL, et al. (1997) Detection of dopaminergic neurotransmitter activity using pharmacologic MRI: Correlation with PET, microdialysis, and behavioral data. Magn Reson Med 38:389–398. doi: 10.1002/mrm.1910380306

Chen YI, Brownell AL, Galpern W, et al. (1999) Detection of dopaminergic cell loss and neural transplantation using pharmacological MRI, PET and behavioral assessment. Neuroreport 10:2881–6.

Chen YI, Choi J-K, Jenkins BG (2005b) Mapping interactions between dopamine and adenosine A2a receptors using pharmacologic MRI. Synapse 55:80–8. doi: 10.1002/syn.20091

Chen YI, Choi J-K, Xu H, et al. (2010) Pharmacologic neuroimaging of the ontogeny of dopamine receptor function. Dev Neurosci 32:125–38. doi: 10.1159/000286215

Chen YI, Famous K, Xu H, et al. (2011) Cocaine self-administration leads to alterations in temporal responses to cocaine challenge in limbic and motor circuitry. Eur J Neurosci 34:800–15. doi: 10.1111/j.1460-9568.2011.07806.x

Chen Z, Silva AC, Yang J, Shen J (2005c) Elevated endogenous GABA level correlates with decreased fMRI signals in the rat brain during acute inhibition of GABA transaminase. J Neurosci Res 79:383–91. doi: 10.1002/jnr.20364

Choi J-K, Chen YI, Hamel E, Jenkins BG (2006a) Brain hemodynamic changes mediated by dopamine receptors: Role of the cerebral microvasculature in dopamine-mediated neurovascular coupling. Neuroimage 30:700–12. doi: 10.1016/j.neuroimage.2005.10.029

Choi J-K, Dedeoglu A, Jenkins BG (2010a) Longitudinal monitoring of motor neuron circuitry in FALS rats using in-vivo phMRI. Neuroreport 21:157–62. doi: 10.1097/WNR.0b013e328330eb9e

Choi J-K, Mandeville JB, Chen YI, et al. (2010b) Imaging brain regional and cortical laminar effects of selective D3 agonists and antagonists. Psychopharmacology (Berl) 212:59–72. doi: 10.1007/s00213-010-1924-6

Choi J-K, Mandeville JB, Chen YI, et al. (2006b) High resolution spatial mapping of nicotine action using pharmacologic magnetic resonance imaging. Synapse 60:152–7. doi: 10.1002/syn.20284

Couch Y, Martin CJ, Howarth C, et al. (2013) Systemic inflammation alters central 5-HT function as determined by pharmacological MRI. Neuroimage 75:177–86. doi: 10.1016/j.neuroimage.2013.02.046

Delfino M, Kalisch R, Czisch M, et al. (2007) Mapping the effects of three dopamine agonists with different dyskinetogenic potential and receptor selectivity using pharmacological functional magnetic resonance imaging. Neuropsychopharmacology 32:1911–21. doi: 10.1038/sj.npp.1301329

Dixon AL, Prior M, Morris PM, et al. (2005) Dopamine antagonist modulation of amphetamine response as detected using pharmacological MRI. Neuropharmacology 48:236–45. doi: 10.1016/j.neuropharm.2004.10.006

Dodd GT, Stark JA, McKie S, et al. (2009) Central cannabinoid signaling mediating food intake: a pharmacological-challenge magnetic resonance imaging and functional histology study in rat. Neuroscience 163:1192–200. doi: 10.1016/j.neuroscience.2009.07.022

Dodd GT, Williams SR, Luckman SM (2010) Functional magnetic resonance imaging and c-Fos mapping in rats following a glucoprivic dose of 2-deoxy-D-glucose. J Neurochem 113:1123–32. doi: 10.1111/j.1471-4159.2010.06671.x

Easton N, Marshall F, Fone K, Marsden C (2007a) Atomoxetine produces changes in cortico-basal thalamic loop circuits: assessed by phMRI BOLD contrast. Neuropharmacology 52:812–26. doi: 10.1016/j.neuropharm.2006.09.024

Easton N, Marshall F, Fone KCF, Marsden CA (2007b) Differential effects of the D- and L- isomers of amphetamine on pharmacological MRI BOLD contrast in the rat. Psychopharmacology (Berl) 193:11–30. doi: 10.1007/s00213-007-0756-5

Easton N, Marshall FH, Marsden CA, Fone KCF (2009) Mapping the central effects of methylphenidate in the rat using pharmacological MRI BOLD contrast. Neuropharmacology 57:653–64. doi: 10.1016/j.neuropharm.2009.08.018

Easton N, Shah YB, Marshall FH, et al. (2006) Guanfacine produces differential effects in frontal cortex compared with striatum: assessed by phMRI BOLD contrast. Psychopharmacology (Berl) 189:369–85. doi: 10.1007/s00213-006-0558-1

Gass N, Schwarz AJ, Sartorius A, et al. (2014) Sub-anesthetic ketamine modulates intrinsic BOLD connectivity within the hippocampal-prefrontal circuit in the rat. Neuropsychopharmacology 39:895–906. doi: 10.1038/npp.2013.290

Governo RJM, Morris PG, Marsden CA, Chapman V (2008) Gabapentin evoked changes in functional activity in nociceptive regions in the brain of the anaesthetized rat: an fMRI study. Br J Pharmacol 153:1558–67. doi: 10.1038/bjp.2008.27

Governo RJM, Morris PG, Prior MJW, et al. (2006) Capsaicin-evoked brain activation and central sensitization in anaesthetised rats: a functional magnetic resonance imaging study. Pain 126:35–45. doi: 10.1016/j.pain.2006.06.012

Gozzi A, Colavito V, Seke Etet PF, et al. (2012) Modulation of fronto-cortical activity by modafinil: a functional imaging and fos study in the rat. Neuropsychopharmacology 37:822–37. doi: 10.1038/npp.2011.260

Gozzi A, Crestan V, Turrini G, et al. (2010) Antagonism at serotonin 5-HT(2A) receptors modulates functional activity of frontohippocampal circuit. Psychopharmacology (Berl) 209:37–50. doi: 10.1007/s00213-009-1772-4

Gozzi A, Herdon H, Schwarz A, et al. (2008a) Pharmacological stimulation of NMDA receptors via co-agonist site suppresses fMRI response to phencyclidine in the rat. Psychopharmacology (Berl) 201:273–84. doi: 10.1007/s00213-008-1271-z

Gozzi A, Large CH, Schwarz A, et al. (2008b) Differential effects of antipsychotic and glutamatergic agents on the phMRI response to phencyclidine. Neuropsychopharmacology 33:1690–703. doi: 10.1038/sj.npp.1301547

Gozzi A, Lepore S, Vicentini E, et al. (2013) Differential effect of orexin-1 and CRF-1 antagonism on stress circuits: a fMRI study in the rat with the pharmacological stressor Yohimbine. Neuropsychopharmacology 38:2120–30. doi: 10.1038/npp.2013.109

Gozzi A, Schwarz A, Reese T, et al. (2006) Region-specific effects of nicotine on brain activity: a pharmacological MRI study in the drug-naïve rat. Neuropsychopharmacology 31:1690–703. doi: 10.1038/sj.npp.1300955

Gozzi A, Schwarz AJ, Reese T, et al. (2005) Functional magnetic resonance mapping of intracerebroventricular infusion of a neuroactive peptide in the anaesthetised rat. J Neurosci Methods 142:115–24. doi: 10.1016/j.jneumeth.2004.08.013

Gozzi A, Tessari M, Dacome L, et al. (2011a) Neuroimaging evidence of altered fronto-cortical and striatal function after prolonged cocaine self-administration in the rat. Neuropsychopharmacology 36:2431–40. doi: 10.1038/npp.2011.129

Gozzi A, Turrini G, Piccoli L, et al. (2011b) Functional magnetic resonance imaging reveals different neural substrates for the effects of orexin-1 and orexin-2 receptor antagonists. PLoS One 6:e16406. doi: 10.1371/journal.pone.0016406

Hackler EA, Byun NE, Jones CK, et al. (2010) Selective potentiation of the metabotropic glutamate receptor subtype 2 blocks phencyclidine-induced hyperlocomotion and brain activation. Neuroscience 168:209–18. doi: 10.1016/j.neuroscience.2010.02.057

Hackler EA, Turner GH, Gresch PJ, et al. (2007) 5-Hydroxytryptamine2C receptor contribution to m-chlorophenylpiperazine and N-methyl-beta-carboline-3-carboxamide-induced anxiety-like behavior and limbic brain activation. J Pharmacol Exp Ther 320:1023–9. doi: 10.1124/jpet.106.113357

Hess A, Sergejeva M, Budinsky L, et al. (2007) Imaging of hyperalgesia in rats by functional MRI. Eur J pain 11:109–19. doi: 10.1016/j.ejpain.2006.01.005

Hewitt KN, Shah YB, Prior MJW, et al. (2005) Behavioural and pharmacological magnetic resonance imaging assessment of the effects of methylphenidate in a potential new rat model of attention deficit hyperactivity disorder. Psychopharmacology (Berl) 180:716–23. doi: 10.1007/s00213-005-2272-9

Hoff EI, Steinbusch HWM, van Oostenbrugge RJ, et al. (2011) Alterations in the cholinergic system after frontal cortical infarction in rat brain: pharmacological magnetic resonance imaging of muscarinic receptor responsiveness and stereological analysis of cholinergic forebrain neurons. Neurobiol Dis 43:625–34. doi: 10.1016/j.nbd.2011.05.011

Hoff EI, van Oostenbrugge RJ, Otte WM, et al. (2010) Pharmacological magnetic resonance imaging of muscarinic acetylcholine receptor activation in rat brain. Neuropharmacology 58:1252–7. doi: 10.1016/j.neuropharm.2010.03.007

Hooker BA, Tobon G, Baker SJ, et al. (2014) Gabapentin-induced pharmacodynamic effects in the spinal nerve ligation model of neuropathic pain. Eur J Pain 18:223–37. doi: 10.1002/j.1532-2149.2013.00364.x

Houston GC, Papadakis NG, Carpenter TA, et al. (2001) Mapping of brain activation in response to pharmacological agents using fMRI in the rat. Magn Reson Imaging 19:905–919. doi: 10.1016/S0730-725X(01)00405-2

Ireland MD, Lowe AS, Reavill C, et al. (2005) Mapping the effects of the selective dopamine D2/D3 receptor agonist quinelorane using pharmacological magnetic resonance imaging. Neuroscience 133:315–26. doi: 10.1016/j.neuroscience.2005.02.018

Jones N, Messenger MJ, O’Neill MJ, et al. (2008) AMPA receptor potentiation can prevent ethanol-induced intoxication. Neuropsychopharmacology 33:1713–23. doi: 10.1038/sj.npp.1301562

Jones N, O’Neill MJ, Tricklebank M, et al. (2005) Examining the neural targets of the AMPA receptor potentiator LY404187 in the rat brain using pharmacological magnetic resonance imaging. Psychopharmacology (Berl) 180:743–51. doi: 10.1007/s00213-005-2254-y

Kalisch R, Delfino M, Murer MG, Auer DP (2005) The phenylephrine blood pressure clamp in pharmacologic magnetic resonance imaging: reduction of systemic confounds and improved detectability of drug-induced BOLD signal changes. Psychopharmacology (Berl) 180:774–80. doi: 10.1007/s00213-005-2252-0

Kalisch R, Salomé N, Platzer S, et al. (2004) High trait anxiety and hyporeactivity to stress of the dorsomedial prefrontal cortex: a combined phMRI and Fos study in rats. Neuroimage 23:382–91. doi: 10.1016/j.neuroimage.2004.06.012

Klomp A, Tremoleda JL, Wylezinska M, et al. (2012) Lasting effects of chronic fluoxetine treatment on the late developing rat brain: age-dependent changes in the serotonergic neurotransmitter system assessed by pharmacological MRI. Neuroimage 59:218–26. doi: 10.1016/j.neuroimage.2011.07.082

Kochanek PM, Hendrich KS, Jackson EK, et al. (2005) Characterization of the effects of adenosine receptor agonists on cerebral blood flow in uninjured and traumatically injured rat brain using continuous arterial spin-labeled magnetic resonance imaging. J Cereb blood flow Metab 25:1596–612. doi: 10.1038/sj.jcbfm.9600154

Kochanek PM, Hendrich KS, Robertson CL, et al. (2001) Assessment of the effect of 2-chloroadenosine in normal rat brain using spin-labeled MRI measurement of perfusion. Magn Reson Med 45:924–9.

Kocsis P, Gajári D, Deli L, et al. (2013) Effect of tolperisone on the resting brain and on evoked responses, an phMRI BOLD study. Brain Res Bull 99:34–40. doi: 10.1016/j.brainresbull.2013.09.008

Kocsis P, Gyertyán I, Éles J, et al. (2014) Vascular action as the primary mechanism of cognitive effects of cholinergic, CNS-acting drugs, a rat phMRI BOLD study. J Cereb Blood Flow Metab 34:995–1000. doi: 10.1038/jcbfm.2014.47

Kuriwaki J-I, Nishijo H, Kondoh T, et al. Comparison of brain activity between dopamine D2 receptor-knockout and wild mice in response to dopamine agonist and antagonist assessed by fMRI. Neurosignals 13:227–40. doi: 10.1159/000079337

Littlewood CL, Cash D, Dixon AL, et al. (2006a) Using the BOLD MR signal to differentiate the stereoisomers of ketamine in the rat. Neuroimage 32:1733–46. doi: 10.1016/j.neuroimage.2006.05.022

Littlewood CL, Jones N, O’Neill MJ, et al. (2006b) Mapping the central effects of ketamine in the rat using pharmacological MRI. Psychopharmacology (Berl) 186:64–81. doi: 10.1007/s00213-006-0344-0

Liu CH, Greve DN, Dai G, et al. (2007) Remifentanil administration reveals biphasic phMRI temporal responses in rat consistent with dynamic receptor regulation. Neuroimage 34:1042–53. doi: 10.1016/j.neuroimage.2006.10.028

Liu X, Yang Z, Li R, et al. (2012) Responses of dopaminergic, serotonergic and noradrenergic networks to acute levo-tetrahydropalmatine administration in naïve rats detected at 9.4 T. Magn Reson Imaging 30:261–70. doi: 10.1016/j.mri.2011.09.006

Lowe AS, Williams SCR, Symms MR, et al. (2002) Functional magnetic resonance neuroimaging of drug dependence: naloxone-precipitated morphine withdrawal. Neuroimage 17:902–10.

Lu H, Chefer S, Kurup PK, et al. (2012) fMRI response in the medial prefrontal cortex predicts cocaine but not sucrose self-administration history. Neuroimage 62:1857–66. doi: 10.1016/j.neuroimage.2012.05.076

Luo F, Schmidt KF, Fox GB, Ferris CF (2009) Differential responses in CBF and CBV to cocaine as measured by fMRI: implications for pharmacological MRI signals derived oxygen metabolism assessment. J Psychiatr Res 43:1018–24. doi: 10.1016/j.jpsychires.2008.11.009

Luo F, Seifert TR, Edalji R, et al. (2008) Non-invasive characterization of beta-amyloid(1-40) vasoactivity by functional magnetic resonance imaging in mice. Neuroscience 155:263–9. doi: 10.1016/j.neuroscience.2008.04.021

Luo F, Wu G, Li Z, Li S-J (2003) Characterization of effects of mean arterial blood pressure induced by cocaine and cocaine methiodide on BOLD signals in rat brain. Magn Reson Med 49:264–70. doi: 10.1002/mrm.10366

Luo F, Xi Z-X, Wu G, et al. (2004) Attenuation of brain response to heroin correlates with the reinstatement of heroin-seeking in rats by fMRI. Neuroimage 22:1328–35. doi: 10.1016/j.neuroimage.2004.03.017

Mandeville JB, Jenkins BG, Kosofsky BE, et al. (2001) Regional sensitivity and coupling of BOLD and CBV changes during stimulation of rat brain. Magn Reson Med 45:443–7.

Marota JJ, Mandeville JB, Weisskoff RM, et al. (2000) Cocaine activation discriminates dopaminergic projections by temporal response: an fMRI study in Rat. Neuroimage 11:13–23. doi: 10.1006/nimg.1999.0520

Mueggler T, Baumann D, Rausch M, Rudin M (2001) Bicuculline-induced brain activation in mice detected by functional magnetic resonance imaging. Magn Reson Med 46:292–8.

Mueggler T, Razoux F, Russig H, et al. (2011) Mapping of CBV changes in 5-HT(1A) terminal fields by functional MRI in the mouse brain. Eur Neuropsychopharmacol 21:344–53. doi: 10.1016/j.euroneuro.2010.06.010

Mueggler T, Sturchler-Pierrat C, Baumann D, et al. (2002) Compromised hemodynamic response in amyloid precursor protein transgenic mice. J Neurosci 22:7218–24. doi: 20026680

Nguyen T V, Brownell AL, Iris Chen YC, et al. (2000) Detection of the effects of dopamine receptor supersensitivity using pharmacological MRI and correlations with PET. Synapse 36:57–65. doi: 10.1002/(SICI)1098-2396(200004)36:1<57::AID-SYN6>3.0.CO;2-K

Nordquist RE, Risterucci C, Moreau JL, et al. (2008) Effects of aripiprazole/OPC-14597 on motor activity, pharmacological models of psychosis, and brain activity in rats. Neuropharmacology 54:405–16. doi: 10.1016/j.neuropharm.2007.10.010

Panizzutti R, Rausch M, Zurbrügg S, et al. The pharmacological stimulation of NMDA receptors via co-agonist site: an fMRI study in the rat brain. Neurosci Lett 380:111–5. doi: 10.1016/j.neulet.2005.01.062

Perles-Barbacaru T-A, Procissi D, Demyanenko A V, et al. (2011) Quantitative pharmacologic MRI: mapping the cerebral blood volume response to cocaine in dopamine transporter knockout mice. Neuroimage 55:622–8. doi: 10.1016/j.neuroimage.2010.12.048

Perles-Barbacaru T-A, Procissi D, Demyanenko A V, Jacobs RE (2012) Quantitative pharmacologic MRI in mice. NMR Biomed 25:498–505. doi: 10.1002/nbm.1760

Preece M, Mukherjee B, Huang CL, et al. (2001) Detection of pharmacologically mediated changes in cerebral activity by functional magnetic resonance imaging: the effects of sulpiride in the brain of the anaesthetised rat. Brain Res 916:107–14.

Preece MA, Sibson NR, Raley JM, et al. (2007) Region-specific effects of a tyrosine-free amino acid mixture on amphetamine-induced changes in BOLD fMRI signal in the rat brain. Synapse 61:925–32. doi: 10.1002/syn.20442

Preece MA, Taylor MJ, Raley J, et al. (2009) Evidence that increased 5-HT release evokes region-specific effects on blood-oxygenation level-dependent functional magnetic resonance imaging responses in the rat brain. Neuroscience 159:751–9. doi: 10.1016/j.neuroscience.2008.12.032

Rausch M, Gentsch C, Enz A, et al. (2005) A study paradigm allowing comparison of multiple high-resolution rCBV-maps for the examination of drug effects. NMR Biomed 18:260–8. doi: 10.1002/nbm.956

Razoux F, Baltes C, Mueggler T, et al. (2013) Functional MRI to assess alterations of functional networks in response to pharmacological or genetic manipulations of the serotonergic system in mice. Neuroimage 74:326–36. doi: 10.1016/j.neuroimage.2013.02.031

Reese T, Bjelke B, Porszasz R, et al. (2000) Regional brain activation by bicuculline visualized by functional magnetic resonance imaging. Time-resolved assessment of bicuculline-induced changes in local cerebral blood volume using an intravascular contrast agent. NMR Biomed 13:43–9.

Reese T, Bochelen D, Baumann D, et al. (2002) Impaired functionality of reperfused brain tissue following short transient focal ischemia in rats. Magn Reson Imaging 20:447–54.

Ren J, Xu H, Choi J-K, et al. (2009) Dopaminergic response to graded dopamine concentration elicited by four amphetamine doses. Synapse 63:764–72. doi: 10.1002/syn.20659

Risterucci C, Jeanneau K, Schöppenthau S, et al. (2005) Functional magnetic resonance imaging reveals similar brain activity changes in two different animal models of schizophrenia. Psychopharmacology (Berl) 180:724–34. doi: 10.1007/s00213-005-2204-8

Roberts TJ, Price J, Williams SCR, Modo M (2007) Pharmacological MRI of stem cell transplants in the 3-nitropropionic acid-damaged striatum. Neuroscience 144:100–9. doi: 10.1016/j.neuroscience.2006.09.015

Roberts TJ, Williams SCR, Modo M (2008) A pharmacological MRI assessment of dizocilpine (MK-801) in the 3-nitroproprionic acid-lesioned rat. Neurosci Lett 444:42–7. doi: 10.1016/j.neulet.2008.07.090

Sánchez-Pernaute R, Jenkins BG, Choi J-K, et al. (2007) In vivo evidence of D3 dopamine receptor sensitization in parkinsonian primates and rodents with l-DOPA-induced dyskinesias. Neurobiol Dis 27:220–7. doi: 10.1016/j.nbd.2007.04.016

Sárvári M, Deli L, Kocsis P, et al. (2014a) Estradiol and isotype-selective estrogen receptor agonists modulate the mesocortical dopaminergic system in gonadectomized female rats. Brain Res 1583:1–11. doi: 10.1016/j.brainres.2014.06.020

Sárvári M, Kocsis P, Deli L, et al. (2014b) Ghrelin modulates the fMRI BOLD response of homeostatic and hedonic brain centers regulating energy balance in the rat. PLoS One 9:e97651. doi: 10.1371/journal.pone.0097651

Schmidt KF, Febo M, Shen Q, et al. (2006) Hemodynamic and metabolic changes induced by cocaine in anesthetized rat observed with multimodal functional MRI. Psychopharmacology (Berl) 185:479–86. doi: 10.1007/s00213-006-0319-1

Schwarz A, Gozzi A, Reese T, et al. (2004a) Selective dopamine D(3) receptor antagonist SB-277011-A potentiates phMRI response to acute amphetamine challenge in the rat brain. Synapse 54:1–10. doi: 10.1002/syn.20055

Schwarz AJ, Gozzi A, Bifone A (2009) Community structure in networks of functional connectivity: resolving functional organization in the rat brain with pharmacological MRI. Neuroimage 47:302–11. doi: 10.1016/j.neuroimage.2009.03.064

Schwarz AJ, Gozzi A, Reese T, et al. (2007a) Pharmacological modulation of functional connectivity: the correlation structure underlying the phMRI response to d-amphetamine modified by selective dopamine D3 receptor antagonist SB277011A. Magn Reson Imaging 25:811–20. doi: 10.1016/j.mri.2007.02.017

Schwarz AJ, Gozzi A, Reese T, Bifone A (2007b) In vivo mapping of functional connectivity in neurotransmitter systems using pharmacological MRI. Neuroimage 34:1627–36. doi: 10.1016/j.neuroimage.2006.11.010

Schwarz AJ, Gozzi A, Reese T, Bifone A (2007c) Functional connectivity in the pharmacologically activated brain: resolving networks of correlated responses to d-amphetamine. Magn Reson Med 57:704–13. doi: 10.1002/mrm.21179

Schwarz AJ, Whitcher B, Gozzi A, et al. (2007d) Study-level wavelet cluster analysis and data-driven signal models in pharmacological MRI. J Neurosci Methods 159:346–60. doi: 10.1016/j.jneumeth.2006.07.017

Schwarz AJ, Zocchi A, Reese T, et al. (2004b) Concurrent pharmacological MRI and in situ microdialysis of cocaine reveal a complex relationship between the central hemodynamic response and local dopamine concentration. Neuroimage 23:296–304. doi: 10.1016/j.neuroimage.2004.05.001

Sekar S, Jonckers E, Verhoye M, et al. (2013) Subchronic memantine induced concurrent functional disconnectivity and altered ultra-structural tissue integrity in the rodent brain: revealed by multimodal MRI. Psychopharmacology (Berl) 227:479–91. doi: 10.1007/s00213-013-2966-3

Sekar S, Van Audekerke J, Vanhoutte G, et al. (2011a) Neuroanatomical targets of reboxetine and bupropion as revealed by pharmacological magnetic resonance imaging. Psychopharmacology (Berl) 217:549–57. doi: 10.1007/s00213-011-2311-7

Sekar S, Verhoye M, Van Audekerke J, et al. (2011b) Neuroadaptive responses to citalopram in rats using pharmacological magnetic resonance imaging. Psychopharmacology (Berl) 213:521–31. doi: 10.1007/s00213-010-2084-4

Shah YB, Haynes L, Prior MJW, et al. (2005) Functional magnetic resonance imaging studies of opioid receptor-mediated modulation of noxious-evoked BOLD contrast in rats. Psychopharmacology (Berl) 180:761–73. doi: 10.1007/s00213-005-2214-6

Shah YB, Prior MJW, Dixon AL, et al. (2004) Detection of cannabinoid agonist evoked increase in BOLD contrast in rats using functional magnetic resonance imaging. Neuropharmacology 46:379–87. doi: 10.1016/j.neuropharm.2003.09.023

Shih Y-YI, Chen Y-Y, Chen C-C V, et al. (2008) Whole-brain functional magnetic resonance imaging mapping of acute nociceptive responses induced by formalin in rats using atlas registration-based event-related analysis. J Neurosci Res 86:1801–11. doi: 10.1002/jnr.21638

Shoaib M, Lowe AS, Williams SCR (2004) Imaging localised dynamic changes in the nucleus accumbens following nicotine withdrawal in rats. Neuroimage 22:847–54. doi: 10.1016/j.neuroimage.2004.01.026

Sloan HL, Austin VC, Blamire AM, et al. (2010) Regional differences in neurovascular coupling in rat brain as determined by fMRI and electrophysiology. Neuroimage 53:399–411. doi: 10.1016/j.neuroimage.2010.07.014

Stark JA, Davies KE, Williams SR, Luckman SM (2006) Functional magnetic resonance imaging and c-Fos mapping in rats following an anorectic dose of m-chlorophenylpiperazine. Neuroimage 31:1228–37. doi: 10.1016/j.neuroimage.2006.01.046

Stark JA, McKie S, Davies KE, et al. (2008) 5-HT(2C) antagonism blocks blood oxygen level-dependent pharmacological-challenge magnetic resonance imaging signal in rat brain areas related to feeding. Eur J Neurosci 27:457–65. doi: 10.1111/j.1460-9568.2007.06002.x

Suarez S V, Amadon A, Giacomini E, et al. (2009) Brain activation by short-term nicotine exposure in anesthetized wild-type and beta2-nicotinic receptors knockout mice: a BOLD fMRI study. Psychopharmacology (Berl) 202:599–610. doi: 10.1007/s00213-008-1338-x

Taketa Y, Niikura K, Kobayashi Y, et al. (2010) Direct evidence for the ongoing brain activation by enhanced dynorphinergic system in the spinal cord under inflammatory noxious stimuli. Anesthesiology 112:418–31. doi: 10.1097/ALN.0b013e3181ca31d9

Tarrasch R, Goelman G, Joel D, et al. (2005) Long-term functional consequences of quinolinic acid striatal lesions and their alteration following an addition of a globus pallidus lesion assessed using pharmacological magnetic resonance imaging. Exp Neurol 196:244–53. doi: 10.1016/j.expneurol.2005.07.023

Upadhyay J, Baker SJ, Rajagovindan R, et al. (2013) Pharmacological modulation of brain activity in a preclinical model of osteoarthritis. Neuroimage 64:341–55. doi: 10.1016/j.neuroimage.2012.08.084

Van der Marel K, Homberg JR, Otte WM, Dijkhuizen RM (2013) Functional and structural neural network characterization of serotonin transporter knockout rats. PLoS One 8:e57780. doi: 10.1371/journal.pone.0057780

Van der Marel K, Klomp A, Meerhoff GF, et al. (2014) Long-term oral methylphenidate treatment in adolescent and adult rats: differential effects on brain morphology and function. Neuropsychopharmacology 39:263–73. doi: 10.1038/npp.2013.169

Wellman PJ, Clifford PS, Rodriguez JA, et al. (2012) Brain reinforcement system function is ghrelin dependent: studies in the rat using pharmacological fMRI and intracranial self-stimulation. Addict Biol 17:908–19. doi: 10.1111/j.1369-1600.2011.00392.x

Whitcher B, Schwarz AJ, Barjat H, et al. (2005) Wavelet-based cluster analysis: data-driven grouping of voxel time courses with application to perfusion-weighted and pharmacological MRI of the rat brain. Neuroimage 24:281–95. doi: 10.1016/j.neuroimage.2004.08.022

Williams JM, Milatovic D, Gore JC, et al. (2010) Chronic exposure to manganese alters brain responses to amphetamine: a pharmacological magnetic resonance imaging study. Toxicol Sci 114:310–22. doi: 10.1093/toxsci/kfq002

Williams JM, Owens WA, Turner GH, et al. (2007) Hypoinsulinemia regulates amphetamine-induced reverse transport of dopamine. PLoS Biol 5:e274. doi: 10.1371/journal.pbio.0050274

Xi Z-X, Wu G, Stein EA, Li S-J (2004) Opiate tolerance by heroin self-administration: an fMRI study in rat. Magn Reson Med 52:108–14. doi: 10.1002/mrm.20119

Xi Z-X, Wu G, Stein EA, Li S-J (2002) GABAergic mechanisms of heroin-induced brain activation assessed with functional MRI. Magn Reson Med 48:838–43. doi: 10.1002/mrm.10282

Xu H, Li SJ, Bodurka J, et al. (2000) Heroin-induced neuronal activation in rat brain assessed by functional MRI. Neuroreport 11:1085–92.

Zuo Y, Lu H, Vaupel DB, et al. (2011) Acute nicotine-induced tachyphylaxis is differentially manifest in the limbic system. Neuropsychopharmacology 36:2498–512. doi: 10.1038/npp.2011.139

| **Reference** | **Species/sex/strain/**  **weight** | **Induction anaesthetic/**  **Dose/Route** | **Maintenance anaesthetic/**  **Dose/Route** | **Mechanical Ventilation** | **Physiological monitoring** | **fMRI measure** |
| --- | --- | --- | --- | --- | --- | --- |
| Baslow et al., 2005 | Mouse/M/C57BL6/ 30g | Isoflurane/2% in 75:22 NO_2_:O_2_/inhalation | Isoflurane/1% in 75:22 NO_2_:O_2_/inhalation | N | Temp | BOLD |
| Bosshard et al., 2012 | Mouse/both/C57BL6, CB_1_^-/-^, *sns-* CB_1_^-/-^/? | Isoflurane/2-3% in 70:30 air:O_2_/inhalation | Isoflurane/1.2% in 70:30 air:O_2_/inhalation | Y | Temp, HR, RR | BOLD |
| Bouet et al., 2012 | Rat/M/Wistar/ adolescents: 35-49g; adults: 275-299g | Isoflurane/5% in 70-75:25-30 N2O:O2/inhalation | Isoflurane/1.5-2% in 70-75:25-30 N2O:O2/inhalation | N | Temp, BP, RR, blood gases, | BOLD |
| Broberg et al., 2013 | Rat/?/Lister Hooded/300±7g; 290±8g | Isoflurane/3% in 70:30 N2:O2/inhalation | Isoflurane/0.8% in 70:30 N_2_:O_2_/inhalation | Y | Temp, BP, HR, RR, blood gases | rCBV |
| Brown et al., 1999 | Rat/M/Fisher 344/250g | Halothane/2% in 66.6:33.3 N_2_O:O_2_/inhalation | Halothane/0.8-1% in 66.6:33.3 N_2_O:O_2_ | N | Temp | CBF |
| Byun et al., 2014 | Rat /M/SD/250-275g | Isoflurane/2% in 1:2 O2:N2O/inhalation | Isoflurane/0.88% in 1:2 O2:N2O /inhalation | Y | Temp, HR, RR, blood gases | rCBV |
| Canese et al., 2009 | Rat/M/S-D/? | Isoflurane/2% in O_2_/inhalation | Isoflurane/1.8% in O_2_/inhalation | N | BP*, HR*, blood gases* | BOLD |
| Canese et al., 2011 | Rat/M/S-D/? | Isoflurane/2% in O_2_/inhalation | Isoflurane/1.8% in O_2_/inhalation | N |  | BOLD |
| Caramia et al., 1998 | Rat/?/Spontaneously hypertensive/? | Pentobarbital/50mg/kg/i.p. | Pentobarbital/10mg/kg/h/i.p. | Y | Temp, BP, RR, blood gases | rCBV |
| Chen et al., 1997 | Rat/?/S-D/? | Halothane/1.5% in N_2_O:O_2_/inhalation | Halothane/1.5% in N_2_O/O_2_/inhalation | N | Temp, BP**, HR**, blood gases | BOLD, rCBV, rCBF |
| Chen et al., 1999 | Rat/F/S-D/? | Halothane/1.5% in N_2_O:O_2_/inhalation | Halothane/1.5% in N_2_O/O_2_/inhalation | N | Temp, blood gases | BOLD, rCBV** |
| Chen et al., 2001 | Rat/?/S-D/? | Halothane/1.5% in N_2_O:O_2_/inhalation | Halothane/1.5% in N_2_O/O_2_/inhalation | N | Temp, blood gases | BOLD, rCBV** |
| Chen et al., 2005a | Rat/M/S-D/250-300g | Halothane/1% in 1:1 N_2_O:O_2_/inhalation | Halothane/1% in 1:1 N_2_O:O_2_/inhalation | N | Temp, BP, blood gases | rCBV |
| Chen et al., 2005b | Rat/M/S-D/250-300g | Halothane/1% in 1:1 N_2_O:O_2_/inhalation | Halothane/1% in 1:1 N_2_O:O_2_/inhalation | N | Temp, BP, blood gases | rCBV |
| Chen et al., 2005c | Rat/M/S-D/185±25g | Isoflurane/1.5% in 70:30 N_2_:O_2_/inhalation | α-chloralose/80mg/kg/i.v. initially, then 27.6mg/kg/h/i.v. | Y | Temp, BP, HR, RR, blood gases | BOLD |
| Chen et al., 2010 | Rat/M/S-D/ 300-450g (adults ); ? (juveniles) | Halothane/1% 1:1 O_2_:air/inhalation | Halothane/1% 1:1 O_2_:air/inhalation | Y*** | Temp, BP, RR***, blood gases | rCBV |
| Chen et al., 2011 | Rat/M/S-D/ 250-300g | Halothane/1.2%/inhalation | Halothane/1.2%/inhalation | Y | Temp, BP, blood gases | rCBV |
| Choi et al., 2006a | Rat/?/?/? | Halothane/1% in 1:1 N_2_O:O_2_/inhalation | Halothane/1% in 1:1 N_2_O:O_2_/inhalation | N | Temp, BP, blood gases | rCBV |
| Choi et al., 2006b | Rat/M/S-D/? | Halothane/1-1.5% O_2_/inhalation | Halothane/1-1.5% O_2_/inhalation | N | Temp, BP†, blood gases† | BOLD, rCBV |
| Choi et al., 2010a | Rat/M/SOD1 G93A transgenic and S-D/? | Halothane/1-1.5% N_2_O:O_2_/inhalation | Halothane/1-1.5% N_2_O:O_2_/inhalation | N | Temp | rCBV |
| Choi et al., 2010b | Rat/M/S-D/ 250-300g | Halothane/1.2% in 1:2 N_2_O:O_2_/inhalation | Halothane/1.2% in 1:2 N_2_O:O_2_/inhalation | N | Temp, BP, blood gases | rCBV |
| Couch et al., 2013 | Rat/M/S-D/ 250-300g | Halothane/2-3% in 60:40 N_2_O:O_2_/inhalation | Halothane/0.8-1% in 60:40 N_2_O:O_2_/inhalation | Y | Temp, BP, RR, blood gases | BOLD |
| Delfino et al., 2007 | Rat/M/Wistar/ 298±41g | Isoflurane/1.5%/inhalation | Isoflurane/1.5%/inhalation | Y | Temp, BP, RR | BOLD |
| Dixon et al., 2005 | Rat/M/S-D/ 250-300g | Halothane/2% in 70:30 N_2_O:O_2_/inhalation | Halothane/1-1.5% in 70:30 N_2_O:O_2_/inhalation | N | Temp, RR | BOLD |
| Dodd et al., 2009 | Rat/M/S-D/ 250±22g | Isoflurane/2-2.5% in O_2_/inhalation | α-chloralose/60mg/kg/i.v. initially, then 30mg/kg/h/i.v. | N | Temp, blood gases* | BOLD |
| Dodd et al., 2010 | Rat/M/S-D/ 260±25g | Isoflurane/2.5% in O_2_/inhalation | α-chloralose/60mg/kg/i.v. initially, then 30mg/kg/h/i.v. | N | Temp, blood gases | BOLD |
| Easton et al., 2006 | Rat/M/S-D/ 200-250g | Isoflurane/3% in 67:33 N_2_O:O_2_/inhalation | Isoflurane/1.75-2% in 67:33 N_2_O:O_2_/inhalation | N | Temp, BP, RR, blood gases | BOLD |
| Easton et al., 2007a | Rat/M/S-D/ 200-250g | Isoflurane/3% in 67:33 N_2_O:O_2_/inhalation | Isoflurane/1.75-2% in 67:33 N_2_O:O_2_/inhalation | N | Temp, BP, RR, blood gases | BOLD |
| Easton et al., 2007b | Rat/M/S-D/ 200-250g | Isoflurane/3% in 67:33 N_2_O:O_2_/inhalation | Isoflurane/1.75-2% in 67:33 N_2_O:O_2_/inhalation | N | Temp, BP, RR, blood gases | BOLD |
| Easton et al., 2009 | Rat/M/S-D/ 200-250g | Isoflurane/3% in 67:33 N_2_O:O_2_/inhalation | Isoflurane/1.75-2% in 67:33 N_2_O:O_2_/inhalation | N | Temp, BP, RR | BOLD |
| Gass et al., 2014 | Rat/M/SD/368-447g | Isoflurane/4% in 70:30 N2:O2 initially, then 2.5%/inhalation | Medetomidine/0.5ml initially, then 0.14mg/kg/h/s.c. | N | HR, RR | BOLD |
| Governo et al., 2006 | Rat/?/S-D/180-200g | Isoflurane/3% in 65:35 N_2_O:O_2_/inhalation | Isoflurane/1.5% in 65:35 N_2_O:O_2_/inhalation | N | Temp, BP, RR, blood gases | BOLD |
| Governo et al., 2008 | Rat/?/S-D/180-200g | Isoflurane/3% in 60:40 N_2_O:O_2_/inhalation | Isoflurane/1.5% in 40:20 N_2_O:O_2_/inhalation | N | Temp, BP, RR, blood gases | BOLD |
| Gozzi et al., 2005 | Rat/M/S-D/ 250-300g | Halothane/3% in 70:30 N_2_:O_2_/inhalation | Halothane/0.8% in 70:30 N_2_:O_2_/inhalation | Y | Temp, BP, HR, RR blood gases | rCBV |
| Gozzi et al., 2006 | Rat/M/S-D/ 250-300g | Halothane/3% in 70:30 N_2_:O_2_/inhalation | Halothane/0.8% in 70:30 N_2_:O_2_/inhalation | Y | Temp, BP, HR, RR, blood gases | rCBV |
| Gozzi et al., 2008a | Rat/M/S-D/293±5g | Halothane/3% in 70:30 N_2_:O_2_/inhalation | Halothane/0.8% in 70:30 N_2_:O_2_/inhalation | Y | Temp, BP, RR, blood gases | rCBV |
| Gozzi et al., 2008b | Rat/M/S-D/ 293±33g | Halothane/3% in 70:30 N_2_:O_2_/inhalation | Halothane/0.8% in 70:30 N_2_:O_2_/inhalation | Y | Temp, BP, RR, blood gases | rCBV |
| Gozzi et al., 2010 | Rat/M/S-D/ 250-350g | Halothane/3% in 70:30 N_2_:O_2_/inhalation | Halothane/0.8% in 70:30 N_2_:O_2_/inhalation | Y | Temp, BP, RR, blood gases | rCBV |
| Gozzi et al., 2011a | Rat/M/Lister Hooded/ 275-300g | Halothane/3% in 70:30 N_2_:O_2_/inhalation | Halothane/0.8% in 70:30 N_2_:O_2_/inhalation | Y | Temp, BP, RR, blood gases | rCBV |
| Gozzi et al., 2011b | Rat/M/S-D/ 250-344g | Halothane/3% in 70:30 N_2_:O_2_/inhalation | Halothane/0.8% in 70:30 N_2_:O_2_/inhalation | Y | Temp, BP, RR, blood gases | rCBV |
| Gozzi et al., 2012 | Rat/M/S-D/ 250-350g | Halothane/3% in 70:30 N_2_:O_2_/inhalation | Halothane/0.8% in 70:30 N_2_:O_2_/inhalation | Y | Temp, BP, RR, blood gases | rCBV |
| Gozzi et al., 2013 | Rat/M/SD/250-350g | Isoflurane/5% in 1:1 O2:N2/inhalation | Isoflurane/1.1% (1.6% in one condition) in 1:1 O2:N2/inhalation | Y | Temp, BP, RR, blood gases | rCBV |
| Hackler et al., 2007 | Rat/M/S-D/? | Isoflurane/2% in 67:33 N_2_O:O_2_/inhalation | Isoflurane/2% in 67:33 N_2_O:O_2_/inhalation | Y | Temp, HR, RR, blood gases | BOLD |
| Hackler et al., 2010 | Rat/M/S-D/ 250-375g | Isoflurane/2% in 67:33 N_2_O:O_2_/inhalation | Isoflurane/2% in 67:33 N_2_O:O_2_/inhalation | Y | Temp, HR, RR, blood gases | BOLD |
| Hess et al., 2007 | Rat/M/Wistar/ ca350g | Isoflurane/5% in medical air/inhalation | Isoflurane/1-2% in medical air/inhalation | N | Temp, RR, blood gases | BOLD |
| Hewitt et al., 2005 | Rat/M/Lister Hooded/? | Halothane/4% in 33.3:66.6 N_2_O:O_2_/inhalation | Halothane/2% in 33.3:66.6 N_2_O:O_2_/inhalation | N | Temp, RR | BOLD |
| Hoff et al., 2010 | Rat/M/Lewis/ 320-380g | Isoflurane/?/inhalation | Isoflurane/3% in 2:1 N_2_O:O_2_/inhalation | Y | Temp, BP, HR, RR, blood gas | BOLD, rCBV |
| Hoff et al., 2011 | Rat/M/Lewis/ 280-320g | Isoflurane/?/inhalation | Isoflurane/3% in 2:1 N_2_O:O_2_/inhalation | Y | Temp, HR, RR, blood gas | rCBV |
| Hooker et al., 2014 | Rat/M/SD/225-250g | Isoflurane/2% in air/inhalation | Isoflurane/2% in air/inhalation | N | Temp | BOLD |
| Houston et al., 2001 | Rat/M/S-D/ 210-310g | Halothane/5% in 70:30 N_2_O:O_2_/inhalation | α-chloralose/80mg/kg/i.v. initially, then 40mg/kg every 90 mins/i.v. | Y | Temp, HR, BP, RR, blood gases | BOLD |
| Ireland et al., 2005 | Rat/M/S-D/250-300g | Halothane/1.7% in O_2_/inhalation | α-chloralose/60mg/kg/i.v. initially, then 30mg/kg/h/i.v. | N | Temp, HR, BP†, RR | BOLD |
| Jones et al., 2005 | Rat/M/S-D/ 295-410g | Isoflurane/4% in 9:1 medical air:O_2_/inhalation | Isoflurane/1.2% in 9:1 medical air:O_2_/inhalation | N | Temp | BOLD |
| Jones et al., 2008 | Rat/M/S-D/ 210-280g | Isoflurane/4% in 9:1 medical air:O_2_/inhalation | Isoflurane/1.2% in 9:1 medical air:O_2_/inhalation | N | Temp | BOLD |
| Kalisch et al., 2004 | Rat/M/outbred high and low anxiety-related behaviour/? | Halothane/1.5% in 60:40 N_2_:O_2_/inhalation | Halothane/0.8% in 60:40 N_2_:O_2_/inhalation | Y | Temp, BP, HR, RR, blood gases | BOLD |
| Kalisch et al., 2005 | Rat/M/Wistar/ 200-254g | Isoflurane/1.5%/inhalation | Isoflurane/1.5%/inhalation | Y | Temp, BP, RR, blood gas | BOLD |
| Klomp et al., 2012 | Rat/M/Wistar/ 50-80g (periadolescents); 290-320g (adults) | Isoflurane/5% in 70:30 N_2_O:O_2_/inhalation | Isoflurane/1.5-2% in70:30 N_2_O:O_2_/inhalation | N | Temp, BP, RR, blood gases | BOLD |
| Kochanek et al., 2001 | Rat/M/S-D/ 371±12g | Isoflurane/4% in O_2_/inhalation | Pentobarbital/37.5 mg/kg/h/i.v.? | Y | Temp, BP, blood gases | CBF |
| Kochanek et al., 2005 | Rat/M/S-D/ 353±24g | Isoflurane/4% in O_2_/inhalation | Pentobarbital/37.5 mg/kg/h/i.v.? | Y | Temp, BP, blood gases | CBF |
| Kocsis et al., 2013 | Rat/ M/Wistar/240-260g | Isoflurane/5% in air/inhalation | Isoflurane/1-1.5% in air/inhalation | Y | Temp, RR | BOLD |
| Kocsis et al., 2014 | Rat/M/Wistar/240-260 | Isoflurane/5% in air/inhalation | Isoflurane/1-1.5% in air/inhalation | Y | Temp, RR | BOLD |
| Kuriwaki et al., 2004 | Mouse/M/D2 receptor KO and WT/28±5g (WT); 27.6±3.6g (D2R KO) | Urethane/1g/kg and α-chloralose/60mg/kg/i.p | N/A | N |  | CBF |
| Littlewood et al., 2006a | Rat/M/S-D/ 306±20 | Isoflurane/4% in 9:1 medical air:O_2_/inhalation | Isoflurane/1.5% in 9:1 medical air:O_2_/inhalation | Y | Temp, HR, RR | BOLD |
| Littlewood et al., 2006b | Rat/M/S-D/ 358±37g | Isoflurane/4% in 9:1 medical air:O_2_/inhalation | Isoflurane/1.5% in 9:1 medical air:O_2_/inhalation | Y | Temp, HR, RR | BOLD |
| Liu et al., 2007 | Rat/?/?/? | Halothane/?/inhalation | Halothane/0.75%/inhalation | Y | BP, HR, RR, blood gases | rCBV |
| Liu et al., 2012 | Rat/M/S-D/ 300-350g | Urethane/1.2g/kg/? | N/A | Y | Temp, BP, RR, blood gases | BOLD |
| Lowe et al., 2002 | Rat/?/Lister Hooded/320±27g | Urethane/1.75g/kg/i.p | N/A | N | Temp†† | BOLD |
| Lu et al., 2012 | Rat/?/Long-Evans/ 400-450g | Isoflurane/2% in 1:1 air:O_2_/inhalation | Propofol/35mg/kg/h/i.v. | Y | Temp, RR, blood gases | rCBV |
| Luo et al., 2003 | Rat/M/S-D/ 300-350g | Urethane/1.2g/kg/? | N/A | Y | Temp, BP, HR, RR | BOLD |
| Luo et al., 2004 | Rat/M/S-D/ 300-350g | Urethane/1.g2/kg/? | N/A | Y | Temp, BP†††, HR†††, RR | BOLD |
| Luo et al., 2008 | Mouse/F/C57BL/6/? | Meditomidine/1mg/kg/i.p and ketamine/75mg/kg/i.p | N/A | N | Temp | rCBV |
| Luo et al., 2009 | Rat/M/S-D/? | Urethane/1.2/kg/i.p. | N/A | Y | Temp, RR, blood gases | CBF, rCBV |
| Mandeville et al., 2001 | Rat/M/S-D/ 306±27g | Halothane/1.5% O_2_/inhalation | Halothane/0.7% in 80:20 air:O_2_/inhalation | Y | Temp, BP, HR, RR, blood gases | BOLD, rCBV |
| Marota et al., 2000 | Rat/M/S-D/ 225-300g | Halothane/1.5% O_2_/inhalation | Halothane/0.7% in 80:20 air:O_2_/inhalation | Y | Temp, BP, HR, RR, blood gases | rCBV |
| Mueggler et al., 2001 | Mouse/?/Hanlbm/NMRI/25-40g | Isoflurane/3% in 2:1 air:O_2_/inhalation | Isoflurane/1.4-1.5% in 2:1 air:O_2_/inhalation | Y | Temp, blood gases | rCBV |
| Mueggler et al., 2002 | Mouse/?/APP23 and wild type controls/? | Isoflurane/3% in 2:1 air:O_2_/inhalation | Isoflurane/1.4% in 2:1 air:O_2_/inhalation | Y | Temp, blood gases | rCBV |
| Mueggler et al., 2011 | Mouse/M/C57BL/6/22-25g | Isoflurane/?/inhalation | Isoflurane/1.5% in 3:1 air:O_2_/inhalation | Y | Temp, blood gases | rCBV |
| Nordquist et al., 2008 | Rat/M/S-D/ 200-400g | Isoflurane/2-2.5% in 1:0.2 O_2_:air/inhalation | Isoflurane/2-2.5% in 1:0.2 O_2_:air/inhalation | N |  | CBF |
| Panizzutti et al., 2005 | Rat/M/Fisher/ 200-250g | Isoflurane/1.2-1.5% in 2:1 air:O_2_/inhalation | Isoflurane/1.2-1.5% in 2:1 air:O_2_/inhalation | N | Temp, blood gases | rCBV |
| Perles-Barbacaru et al., 2011 | Mouse/?/Dopamine transporter KO and WT + C57BL/6/? | Isoflurane/1.5-1.7% in 70:30 N_2_:O_2_/inhalation | Isoflurane/1.5-1.7% in 70:30 N_2_:O_2_/inhalation | N | Temp, HR, RR | rCBV |
| Perles-Barbacaru et al., 2012 | Mouse/F/C57BL/6/? | Isoflurane/1.5-1.7% in 70:30 N_2_:O_2_/inhalation | Isoflurane/1.5-1.7% in 70:30 N_2_:O_2_/inhalation | N | Temp, HR, RR | rCBV |
| Preece et al., 2001 | Rat/M/S-D/ 270-320g | Halothane/5% in 70:30 N_2_:O_2_/inhalation | α-chloralose/50mg/kg/i.v. initially, then 40mg/kg/h/i.v. | Y | Temp, BP, RR, blood gases | BOLD |
| Preece et al., 2007 | Rat/M/S-D/ 270-320g | Halothane/5% in 60:40 N_2_:O_2_/inhalation | Halothane/1-1.5% in 60:40 N_2_:O_2_/inhalation | Y | Temp, BP, RR, blood gases | BOLD |
| Preece et al., 2009 | Rat/M/S-D/ 250-320g | Halothane/5% in 60:40 N_2_:O_2_/inhalation | Halothane/1-1.5% in 60:40 N_2_:O_2_/inhalation | Y | Temp, BP, RR, blood gases | BOLD |
| Rausch et al., 2005 | Rat/M/S-D/ 200-240g | Isoflurane/2% in 2:1 N_2_O:O_2_/inhalation | Isoflurane/1.2% in 2:1 N_2_O:O_2_/inhalation | Y† | Temp, RR, blood gases† | rCBV |
| Razoux et al., 2013 | Mouse/M/C57BL/6 and 5-HT_1A_R KO and WT/22-25g | Isoflurane/?/inhalation | Isoflurane/1.5% in 3:1 air:O_2_/inhalation | Y | Temp, RR | rCBV |
| Reese et al., 2000 | Rat/M/spontaneously hypertensive/ 220-250g | Isoflurane/1-1.5% in 1:2 O_2_:N_2_O/inhalation | Isoflurane/1-1.5% in 1:2 O_2_:N_2_O/inhalation | Y | Temp, BP, RR | rCBV |
| Reese et al., 2002 | Rat/M/Fisher 344/ 220-250g | Isoflurane/2% in 1:2 O_2_:N_2_O/inhalation | Isoflurane/1-1.5% in 1:2 O_2_:N_2_O/inhalation | Y | Temp, BP, RR, blood gases | rCBV |
| Ren et al., 2009 | Rat/M/S-D/ 300-400g | Halothane/1% in 1:1 N_2_O:O_2_/inhalation | Halothane/1% in 1:1 N_2_O:O_2_/inhalation | N | Temp, BP, blood gases | rCBV |
| Risterucci et al., 2005 | Rat/M/Wistar/ 350-400g | Isoflurane/2-2.5% in 1:0.2 air:O_2_/inhalation | Isoflurane/2-2.5% in 1:0.2 air:O_2_/inhalation | N | Temp, RR, blood gas? | CBF |
| Roberts et al., 2007 | Rat/M/Lewis/? | Isoflurane/4% in 70:30 N_2_O:O_2_/inhalation | Isoflurane/1.2% in 70:30 N_2_O:O_2_/inhalation | N | Temp, BP, HR, RR, blood gases | BOLD |
| Roberts et al., 2008 | Rat/M/Lewis/? | Isoflurane/4% in 70:30 N_2_O:O_2_/inhalation | Isoflurane/1.2% in 70:30 N_2_O:O_2_/inhalation | N | Temp, BP, HR, RR | BOLD |
| Sanchez-Pernaute et al., 2007 | Rat/F/S-D/ 200-250g | Halothane/1% in 1:1 NO_2_‡:O_2_/inhalation | Halothane/1% in 1:1 NO_2_‡:O_2_/inhalation | N | Temp, blood gas | rCBV |
| Sárvári et al., 2014a | Rat /F/ Wistar/? | Isoflurane/5% in air/inhalation | Isoflurane/1-1.6% in air/inhalation | Y | Temp | BOLD |
| Sárvári et al., 2014b | Rat/M+F/Wistar/240-260 (M), 170-190g (F) | Isoflurane/5% in air/inhalation | Isoflurane/1-1.5% in air/inhalation | Y | Temp | BOLD |
| Schmidt et al., 2006 | Rat/M/S-D/ 300-375g | Isoflurane/2.5%/inhalation | Isoflurane/1.1%/inhalation | N | BP, HR, RR | BOLD, CBF |
| Schwarz et al., 2004a | Rat/M/S-D/ 250-350g | Halothane/3% in 70:30 N_2_:O_2_/inhalation | Halothane 0.8% in 70:30 N_2_:O_2_/inhalation | Y | Temp, BP, HR, RR, blood gas | rCBV |
| Schwarz et al., 2004b | Rat/?/?/? | Halothane/3% in 70:30 N_2_:O_2_/inhalation | Halothane 0.8% in 70:30 N_2_:O_2_/inhalation | Y | Temp, BP, HR, RR, blood gas | rCBV |
| Schwarz et al., 2007a | Rat/M/S-D/ 289±6g | Halothane/3% in 70:30 N_2_:O_2_/inhalation | Halothane 0.8% in 70:30 N_2_:O_2_/inhalation | Y | Temp, BP, HR, RR, blood gas | rCBV |
| Schwarz et al., 2007b | Rat/M/S-D/ 287±5g | Halothane/3% in 70:30 N_2_:O_2_/inhalation | Halothane 0.8% in 70:30 N_2_:O_2_/inhalation | Y | Temp, BP, HR, RR, blood gas | rCBV |
| Schwarz et al., 2007c | Rat/M/S-D/? | Halothane/3% in 70:30 N_2_:O_2_/inhalation | Halothane 0.8% in 70:30 N_2_:O_2_/inhalation | Y | Temp, BP, HR, RR, blood gas | rCBV |
| Schwarz et al., 2007d | Rat/M/S-D/ 250-350g | Halothane/3% in 70:30 N_2_:O_2_/inhalation | Halothane 0.8% in 70:30 N_2_:O_2_/inhalation | Y | Temp, BP, HR, RR, blood gas | rCBV |
| Schwarz et al., 2009 | Rat/M/S-D/?/? | Halothane/3% in 70:30 N_2_:O_2_/inhalation | Halothane 0.8% in 70:30 N_2_:O_2_/inhalation | Y | Temp, BP, HR, RR, blood gas | rCBV |
| Sekar et al., 2011a | Rat/M/Lister Hooded/ 260-320g (acute); 350-400g (chronic) | Isoflurane/3.5% in 3:2 N_2_:O_2_/inhalation | Isoflurane/0.9-2% in 3:2 N_2_:O_2_/inhalation | N | Temp, RR | BOLD |
| Sekar et al., 2011b | Rat/M/Lister Hooded/ 260-320g (acute); 350-400g (chronic) | Isoflurane/3.5% in 3:2 N_2_:O_2_/inhalation | Isoflurane/0.9-2% in 3:2 N_2_:O_2_/inhalation | N | Temp, RR | BOLD |
| Sekar et al., 2013 | Rat/M/Lister Hooded/240-300g | Isoflurane/3.5% in 70:30 N_2_:O_2_/inhalation | Isoflurane/0.9-2% in 70:30 N_2_:O_2_/inhalation | N | Temp, RR | BOLD |
| Shah et al., 2004 | Rat/M/Lister Hooded/300-350g | Chloral hydrate/500mg/kg/i.p. | Chloral hydrate/150mg/kg prior to imaging/i.p. | N | Temp, BP* | BOLD |
| Shah et al., 2005 | Rat/M/S-D/ 250-300g | Halothane/? in 70:30 in N_2_O:O_2_/inhalation | Halothane/1-2% in 70:30 N_2_O:O_2_/inhalation | N | Temp, BP* | BOLD |
| Shih et al., 2008 | Rat/M/Wistar/ 250-300g | α-chloralose/70mg/kg/i.p. | N/A | Y | Temp, HR*, blood gas* | BOLD |
| Shoaib et al., 2004 | Rat/M/Lister Hooded/320±27g | Urethane/1.5g/kg/i.p. | N/A | N | Temp | BOLD |
| Sloan et al., 2010 | Rat/?/?/? | Halothane/2-3% in 60:40 N_2_O:O_2_/inhalation | Halothane/0.8% in 60:40 N_2_O:O_2_/inhalation | Y | Temp, BP, RR blood gas | BOLD, rCBV |
| Stark et al., 2006 | Rat/M/S-D/ 330±40g | Halothane/2-3% in O_2_/inhalation | α-chloralose/60mg/kg/i.v. initially, then 30mg/kg/h/i.v. | N | Temp, RR | BOLD |
| Stark et al., 2008 | Rat/M/S-D/ 300±35g | Halothane/2-3% in O_2_/inhalation | α-chloralose/60mg/kg/i.v. initially, then 30mg/kg/h/i.v. | N | Temp, RR | BOLD |
| Suarez et al., 2009 | Mouse/M/β2 receptor KO and C57BL/6 WT/? | 2.5% xylazine + 15% ketamine/0.1mL/10g/i.p. | 2.5% xylazine + 15% ketamine/0.1mL/10g/h/i.p. | N | Temp*, HR, RR*, blood gas* | BOLD |
| Taketa et al., 2010 | Mouse/M/prodynorphin KO and C57BL/6 and 129/SvJ WT/18-23g | Isoflurane/?/inhalation | Isoflurane/1%/inhalation | N |  | BOLD |
| Tarrasch et al., 2005 | Rat/M/Wistar/ 400-500g | Pentobarital/40 mg/kg/i.p. | N/A | N |  | BOLD |
| Upadhyay et al., 2013 | Rat/M/Lewis/ 225-250g | Isoflurane/2% in air/inhalation | Isoflurane/2% in air/inhalation | N | Temp | BOLD |
| Van der Marel et al., 2013 | Rat/M/5-HT transporter KO and Wistar WT/492.6±24.2g (KO); 557.8±76.5g (WT) | Isoflurane/5% in 2:1 air:O_2_/inhalation | Isoflurane/1% in 2:1 air:O_2_/inhalation | Y | Temp, BP, HR, RR, blood gas | BOLD |
| Van der Marel et al., 2014 | Rat /M/Wistar/? | ? | Propofol/60mg/kg/h/i.v. | Y | Temp, HR, RR, blood gases | BOLD |
| Wellman et al., 2011 | Rat/M/S-D/ 225-250g | Halothane/3% in 70:30 N_2_:O_2_/inhalation | Halothane/0.8% in 70:30 N_2_:O_2_/inhalation | Y | Temp, BP, HR, RR, blood gases | rCBV |
| Whitcher et al., 2005 | Rat/?/?/? | Halothane/3% in 70:30 N_2_:O_2_/inhalation | Halothane/0.8% in 70:30 N_2_:O_2_/inhalation | Y | Temp, blood gas | rCBV |
| Williams et al., 2007 | Rat/M/S-D/ 275-350g | Isoflurane/?/inhalation | Isoflurane/0.88%/inhalation | Y | Temp, HR, blood gas | BOLD |
| Williams et al., 2010 | Rat/M/S-D/300g | Isoflurane/2% in O_2_/inhalation | Isoflurane/0.8% in 70:30 N_2_:O_2_/inhalation | Y | Temp, BP†, HR, RR, blood gas | BOLD |
| Xi et al., 2002 | Rat/M/S-D/ 250-350g | Urethane/1.2g/kg/? | Urethane/0.2/0.3g/kg/? | Y | Temp, RR, blood gas* | BOLD |
| Xi et al., 2004 | Rat/M/S-D/ 300-350g | Urethane/1.2g/kg/? | Urethane/0.2/0.3g/kg/? | Y | Temp, BP, HR, RR, blood gas | BOLD |
| Xu et al., 2000 | Rat/M/S-D/ 300-380g | Urethane/1.2g/kg/? | Urethane/0.2/0.3g/kg/? | Y† | BP*, RR, blood gas* | BOLD |
| Zuo et al., 2011 | Rat/M/S-D/ 300-380g | Isoflurane/2% in 1:1 air:O_2_/inhalation | Isoflurane/1.7-1.8% in 70:30 air:O_2_/inhalation | Y | Temp, BP, HR, RR, blood gas | rCBV |

**Table S1**: Summary of details from studies included in review

Abbreviations: M = male, F = female, S-D = Sprague-Dawley, KO = knockout, WT = wild type, temp = temperature, BP = blood pressure, HR = heart rate, RR = respiration rate, BOLD = blood oxygen level dependent, rCBV = relative cerebral blood volume, rCBF = relative cerebral blood flow

* separate animals

** control group only

*** experimental group only

† subset of animals

†† measured at start and end of scan only

††† separate animals, data not presented

‡ presumed typo as inconsistent with cited reference, presume authors intend N_2_O instead

| **Journal** | **Number of Studies** | **Percentage** |
| --- | --- | --- |
| NeuroImage | 21 | 16.7 |
| Psychopharmacology | 19 | 15.1 |
| Neuropsychopharmacology | 11 | 8.7 |
| Magnetic Resonance in Medicine | 9 | 7.1 |
| Neuropharmacology | 6 | 4.8 |
| Neuroscience | 6 | 4.8 |
| Synapse | 5 | 4.0 |
| Magnetic Resonance Imaging | 4 | 3.2 |
| Neuroreport | 3 | 2.4 |
| NMR in Biomedicine | 3 | 2.4 |
| PLoS One | 3 | 2.4 |
| Others (2 papers or fewer) | 36 | 28.6 |
|  |  |  |
| *Total:* | *126* | *100* |

**Table S2**: List of journals publishing phMRI studies eligible for inclusion
